# Effect of Synbiotics on Hygienic Quality of Feed and Pork

**DOI:** 10.3390/ani16060933

**Published:** 2026-03-16

**Authors:** Elżbieta Kukier, Łukasz Bocian, Monika Pytka, Katarzyna Śliżewska

**Affiliations:** 1 School of Medical & Health Sciences, VIZJA University, 59 Okopowa Street, 01-043 Warsaw, Poland; 2 Department of Research Support, National Veterinary Research Institute, 57 Partyzantów Avenue, 24-100 Pulawy, Poland; lukasz.bocian@piwet.pulawy.pl; 3 Department of Biotechnology, Microbiology and Human Nutrition, University of Life Sciences, Skromna 8, 20-704 Lublin, Poland; monika.pytka@up.lublin.pl; 4 Institute of Fermentation Technology and Microbiology, Lodz University of Technology, 171/173 Wólczańska Street, 90-924 Łódź, Poland; katarzyna.slizewska@p.lodz.pl

**Keywords:** synbiotic, feed, pork, hygiene, microorganism

## Abstract

The study aimed to determine the effect of newly developed multi-strain synbiotics on the hygienic quality of feed for pigs and pork edible raw materials originating from animals supplemented with synbiotics. The trial was conducted on 54 feed samples and 54 pigs. Animals were divided into six groups corresponding to three synbiotic preparations (A, B, C), two positive controls with probiotics (D, E), and a negative control (K) with no feed additive. The supplementation of the sow’s basal diet with the feed additive started 10 days before farrowing and continued for 38 days, covering the lactation period. The diet of piglets from groups A-E was supplemented with the feed additive starting from two weeks of age until slaughter at 24 weeks of age. Feed and animal raw materials were tested for the presence of *Salmonella* spp., *Campylobacter* spp., *L. monocytogenes*, *Clostridium* spp., *C. perfringens*, *C. botulinum*, and the count of AMB, TPC, fungi, ASFB, *C. perfringens*, Enterobacteriaceae family, *E. coli*, presumptive *B. cereus*, CoPS, HS, LAB, yeast probiotic strains, and *Enterococcus* spp. Statistically significant differences were found between individual groups in the count of *C. perfringens*, AMB, TPC, *Enterococcus* spp., and LAB in all feeds tested. Moreover, synbiotics A, B, and C lowered the count of AMB, TPC, and LAB, and synbiotics A and C decreased Enterobacteriaceae family contamination in both total raw materials as well as raw materials of fatteners. The higher effectiveness of synbiotics was associated with a higher number of probiotic strains in one preparation. Our study found that dietary supplementation of synbiotics alters the occurrence of *C. perfringens* in feed and enhances the hygienic quality of edible pork raw materials.

## Simple Summary

The study aimed to assess the effect of newly developed multi-strain synbiotics on the hygienic quality of feed for pigs and pork edible raw materials originating from supplemented animals. The bioassay was carried out in six groups corresponding to three synbiotic preparations (A, B, C), two positive controls (probiotics), and a negative control. The sows’ basal diet was supplemented with a feed additive before farrowing and during lactation. The diet of piglets was supplemented starting from two weeks of age until slaughter. Feed and animal raw materials were tested for the presence of *Salmonella*, *Campylobacter*, *L. monocytogenes*, *Clostridium*, *C. perfringens*, *C. botulinum*, and the count of AMB, TPC, fungi, ASFB, *C. perfringens*, Enterobacteriaceae family, *E. coli*, presumptive *B. cereus*, CoPS, HS, LAB, yeast probiotic strains, and *Enterococcus*. Statistically significant differences were found between individual groups in the count of *C. perfringens*, AMB, TPC, *Enterococcus* spp., and LAB in all feed. Synbiotics A, B, and C reduced the count of AMB, TPC, and LAB, and synbiotics A and C decreased Enterobacteriaceae family contamination in both total raw materials and raw materials of fatteners. Our study demonstrates that synbiotics alter *C. perfringens* occurrence in feed and reduce the microbial load of pork.

## 1. Introduction

The beneficial effects of probiotics on gut microflora have been known for decades [1]. The significance of these microbial interventions has grown significantly since the European Union-wide ban on the use of antibiotic growth promoters in 2006 aimed at tackling the emergence of antimicrobial-resistant microbes. In response, the International Scientific Association for Probiotics and Prebiotics defined synbiotics in 2019 as “a mixture comprising live microorganisms and substrate(s) selectively utilized by host microorganisms that confers a health benefit on the host” [2]. A mixture of both live probiotics and prebiotics is considered more effective and longer-lasting than either component alone due to their synergistic action.

To date, the positive effects of synbiotics on health, disease resistance capacity, animal growth performance, feed utilization, fecal enzyme activity, intestinal microbiota, physical quality, and antioxidant properties of meat have been revealed [3,4,5,6,7,8,9,10,11,12]. It has been shown that synbiotics can stimulate and increase the survival of probiotic and autochthonous-specific strains in the intestinal tract and improve the survival of beneficial microbes added to food or feed [13]. Other studies have demonstrated that synbiotics change intestinal structure and permeability, enhance animal immune parameters, reduce the prevalence of microbial hazards in intestinal content, and lower carcass load by pathogens [14,15,16,17,18,19].

Since it became known that the intestinal immune system needs microbial stimulation for proper development and regulation, one strategy involves manipulating the gastrointestinal microbiota through the supplementation of probiotics or synbiotics to obtain a better balance of intestinal flora due to microbial diversity, microbial stability, the presence of metabolites, and interactions with the enteric epithelium and the immune system [20].

In turn, the crucial importance of primary production involves heat-resistant spores of anaerobic *Clostridium*, aerobic *Bacillus* species, and hygienic indicators, as their count directly translates into the probability of pathogen occurrence. The care for good hygienic quality concerns, aside from ready-to-eat food, the edible animal raw materials, as well as feed intended for slaughter animals. Feed microflora could be a source of animal infection, and the well-being of food animals includes quality hygienic feed free of pathogens with a low count of spoiling microbes. Existing pathogen control strategies in animal feed production primarily rely on physical and chemical methods. Physical methods include thermal processing techniques like expansion, extrusion, conditioning, and pelleting, which aim to reduce initial microbial loads [21]. However, these processes can be aggressive to nutritional quality, and recontamination can easily occur during subsequent cooling and storage phases. Chemical interventions involve the use of additives such as organic acids, essential oils, or formaldehyde-based products, which can provide residual protection but may come with concerns regarding specialized application equipment, cost, and potential hazards to worker health [22,23].

Given that microorganisms are ubiquitous, microbial contamination is a global public health concern. Microbial agents can invade the food production chain at any point, with feed microflora being a potential source of animal infection. In 2022, the first and second most reported zoonoses in humans were campylobacteriosis and salmonellosis, respectively, followed by yersiniosis, Shiga toxin-producing *Escherichia* (*E*.) *coli*, and *Listeria* (*L*.) *monocytogenes* infections [24]. Additionally, according to the United Nations Food and Agriculture Organization, pork is the most widely eaten meat in the world at 36%, followed by poultry and beef, and the European Union was the world’s second-largest producer of pork in 2020 [25]. Additionally, a recent study has found livestock meat, specifically pork, to be the most common meat source of foodborne illness outbreaks [26]. The microbiological status of animal raw materials is the sum of primary native contamination due to agonal spread of microorganisms and postmortem migration of intestinal bacteria to extra-intestinal compartments such as mesenteric lymph nodes, spleen, liver, kidney, and cardiac blood within a few minutes after the death of the organism, and secondary external contaminants [27].

While a variety of synbiotic benefits have been observed, there is a notable gap in research regarding the direct impact of these additives on the feed microbiome itself and their subsequent effect on the microbial load of pork edible raw materials. Therefore, this study was designed to fill this knowledge gap by examining the effect of newly developed synbiotics on the hygienic quality of feed for pigs and the derived pork edible raw materials. We hypothesized that synbiotic supplementation would effectively reduce microbial load and pathogen prevalence in feed and pork raw materials compared to both probiotic and non-supplemented controls.

## 2. Materials and Methods

### 2.1. Animal Study

The animal trial was conducted between December and June in a farrow-to-finish herd with 100 genetically similar DanBred sows on a private breeding farm located in the Lubelskie Voivodeship, in the eastern part of Poland. The animals were kept in high-investment indoor facilities, and an all-in-all-out production system with a thorough cleaning and disinfecting procedure between new batches of animals was applied. The animals were housed in fully slatted pig housing systems, with more than 1.00 square meters per pig, a ventilation rate of 0.8 cubic meters per kilogram of body weight per hour, and a temperature of 20–26 °C. In the animal rooms, a light–dark cycle of 12 h of light and 12 h of dark was set. The environmental conditions were the same for each animal group during the trial. The farm met the legal requirements for animal welfare on pig farms in the EU (Council Directive 2008/120/EC of 18 December 2008). The health status of the herd was regularly surveyed, and animals were diagnosed as healthy before the experiment based on clinical, serological, and pathological examinations [15].

An experiment was designed to evaluate the effect of newly developed synbiotics on the hygienic quality of feed intended for pigs and pork raw materials derived from these animals. The study comprised 54 feed samples and 54 pigs (females and castrated males) originating from 30 sows. Three synbiotic preparations (A, B, C) were analyzed in parallel with two probiotics as positive controls (D, E) and a negative control (K) with no feed additive. The criteria for animal selection for the experiment was the weight of sows estimated at 256.7 ± 16.4 kg and in parities 3 to 5. The sows were distributed among 6 groups according to the type of feed additive, with 5 sows each. The basal diet shown in Table 1 was supplemented with feed additives starting 10 days before farrowing and continued for 38 days, covering the lactation period [15].

Piglets were weaned at 28 days of age and were divided into six analogous groups (nine animals each) corresponding to three synbiotics, two probiotics, as well as a negative control group (A–E, K). Piglets born from sows of the negative control group were fed no supplement. Each group of piglets originated from two litters of sows fed the same feed additive. The diet of groups A–E piglets was supplemented with a feed additive starting at two weeks of age until slaughter (24 weeks) at a dose of 0.5 kg per tonne of feed. The mixing of feed additive with the basal diet took place as needed with a mixing device. Animals of trial and control groups were housed and managed consistently during the study.

Feed samples were taken on the 1st, 24th, 38th, 39th, 52nd, 57th, 76th, 77th, 123rd, and 175th days of the experiment, starting with the feed additive start date (10 days before farrowing) as the first day of the trial. Sampling of feed took place immediately after supplementation with the feed additive. Once the feed was sampled, it was transferred within one hour at ambient temperature to the laboratory, where it was frozen at −20 °C until testing. In total, 18 samples of feed for sows, 18 feed samples for weaners, and 18 feed samples for fattening pigs were tested.

The finishers were fasted for 12 h and transported 35 km to a commercial abattoir, where they were allowed to rest. The journey of animals to the slaughterhouse took no more than 40 min. Pre-slaughter feed withdrawal was not applied to weaned piglets. The animals were transported in accordance with the requirements of Council Regulation (EC) No 1/2005 of 22 December 2004 on the protection of animals during transport and related operations, and amending Directives 64/432/EEC and 93/119/EC and Regulation (EC) No 1255/97. At the abattoir, pigs were electrically stunned, then slaughtered by being suspended vertically, bled out through the neck tissue, and scalded at 65 °C according to standard commercial procedures.

The animal raw materials, including gluteal muscles and internal organs, such as the liver and kidney, were sampled from 18 piglets that were 28 days old and 36 fattening pigs that were 165 days old. The animal materials were delivered to the laboratory within one hour in boxes containing ice and frozen at −20 °C to impede enzyme and microbial activity until analysis [28].

All samples in the study were collected under aseptic conditions with sterile instruments and placed into sterile plastic bags, following the guidelines specified in the standard PN-EN ISO 13307:2013-06 [29]. The staff of the Department of Swine Diseases at the National Veterinary Research Institute (NVRI) in Pulawy oversaw the rearing of the herd, collected, and delivered the samples to the laboratory. The animal experiment was approved by the 2nd Local Ethics Committee for Animal Experiments in Lublin (Poland) based on resolution no. 4/2015.

### 2.2. Feed Additives

Three newly developed multi-strain and multi-species synbiotics intended for food-producing animals were assessed in comparison with two commercial probiotics and a blank test (Table 2). The synbiotic preparations were designed by the Institute of Fermentation Technology and Microbiology at the Lodz University of Technology and comprised *Saccharomyces cerevisiae* in an amount of 10^7^ cfu/g, *Lactobacillus* spp. strains in an amount of 10^9^ cfu/g, and 20 g/kg of inulin prebiotic. The probiotic properties and the safety evaluation of the selected strains incorporated into the new synbiotic preparations were previously evidenced and described by the authors [4,30]. The preparation BioPlus_2B^®^ with two *Bacillus* probiotic strains at least 1.6 × 10^10^ cfu/g each (Chr. Hansen A/S, Horsholm, Denmark) and Cylactin^®^_LBC_ME10 with an *Enterococcus* probiotic strain in concentrations of 1 × 10^10^ cfu/g (DSM Nutritional Products Ltd., Kaiseraugst, Switzerland) were used as the reference feed additives (positive controls). A negative control was run in parallel to the synbiotic and probiotic groups with the same procedure except for feed additive supplementation.

### 2.3. Microbiological Analyses

Sixteen hours prior to testing, the samples were thawed at a temperature of 2–8 °C. Preparation of samples, initial suspension, and decimal dilutions for examination was carried out in accordance with the rules described in PN-EN ISO 6887-4:2017-05 [31]. Due to the lack of available literature data on the impact of synbiotics on feed hygiene quality, the widest possible range of indicators was used. For microbiological analyses, standard culture methods were applied to detect the presence of *Salmonella* spp., *Campylobacter* spp., *L. monocytogenes*, *Clostridium* spp., *C. perfringens*, and *C. botulinum*, and to enumerate the total plate count (TPC), aerobic mesophilic bacteria (AMB), fungi, anaerobic spore-forming bacteria (ASFB), *C. perfringens*, the Enterobacteriaceae family, *Escherichia* (*E.*) *coli*, presumptive *B. cereus*, coagulase-positive staphylococci (CoPS), hemolytic streptococci (HS), mesophilic lactic acid bacteria (LAB), yeast probiotic strains, and *Enterococcus* spp. (Table 3). Results of qualitative analyses (presence of microorganisms) were expressed as prevalence in percentage. All quantitative analyses (count) were carried out in duplicate and calculated in colony-forming units per gram (cfu/g) and then log_10_ transformed to obtain log-normally distributed data prior to statistical analysis. To validate the test conditions in the laboratory, both negative and positive controls were carried out simultaneously using reference strains.

The microbial status of the negative controls of feed and animal raw materials is shown in Table 4. Microbiological analyses were conducted in the biosafety level 2 laboratory, which has access control, at the Department of Hygiene of Animal Feedingstuffs at NVRI in Pulawy.

### 2.4. Typing of Clostridium spp. Isolates by PCR Methods

Presumptive isolates of *C. perfringens* obtained from a standard culture method were subsequently confirmed with a multiplex polymerase chain reaction method. The presence of the *cpa* (α toxin) gene, recognized as species identification, as well as the *cpb* (β), *cpb2* (β2), *etx* (ε), *iap* (ι), and *cpe* (enterotoxin) genes, were detected following the protocol of Baums et al. [47], as modified by Kukier et al. [48]. The change involved the extraction where the template DNA was obtained from a thermolyzed overnight culture of *C. perfringens* on Willis–Hobbs agar, previously incubated at 37 °C in anaerobic conditions. To produce an anaerobic atmosphere in a jar the Oxoid Anaero*Gen* (Oxoid Ltd., Basingstoke, UK) was used. The isolates suspected to be botulinum neurotoxin (BoNT)-producing *Clostridia* were verified by a real-time PCR assay, where the nontoxic nonhemagglutinin-encoding gene (*ntnh*), distinctive to all BoNT-producing *Clostridia*, was detected [49].

### 2.5. Calculations and Statistics

The statistical analysis included results obtained from a total of 54 feed samples. Feed samples intended for sows, weaners, and fatteners were analyzed separately, with 18 samples in each category. Within each category, 3 feed samples originated from each of the experimental groups (A–E, K). For animal raw materials, the analysis was conducted on the results obtained from 162 samples. Fifty-four samples originated from piglets and represented 18 animals, with three samples collected from each animal (kidney, liver, and muscle). Each experimental group consisted of three animals, resulting in a total of nine individual raw material samples per group. An analogous analysis was performed for samples collected from fatteners, for which the number of animals was twice as high, i.e., six animals (18 raw material samples) per experimental group. Several statistical tests were applied to evaluate the effect of dietary supplementation on the occurrence of microbiological agents. Dichotomous qualitative data were analyzed using the chi-square test with appropriate corrections (Fisher’s exact test, Yates’ correction, or chi-square test), depending on group size and result distribution. Quantitative data were analyzed using non-parametric methods due to limited group sizes and the lack of normal distribution within individual groups, which was formally assessed using the Shapiro–Wilk test. The Kruskal–Wallis test was applied to detect statistically significant differences among all experimental groups. Additionally, the Mann–Whitney U test was used to compare groups A-E with the negative control group K. Each microbiological parameter was analyzed independently. For all analyses, the significance level was set at α = 0.05. Statistical calculations were performed using TIBCO Software Inc., Palo Alto, CA, USA (2017), Statistica (data analysis software system), version 13.

## 3. Results

### 3.1. Feed

The analysis of all feeds identified a correlation between *C. perfringens* occurrence and the type of feed additive (Table 5). A statistically significant difference was found between groups C and K (*p* = 0.0498), as well as between groups D and K (*p* = 0.0498). There was no statistically significant difference for the other groups in the trial regarding *C. perfringens* nor for the occurrence of *Listeria* spp. or *Clostridium* spp.

**Figure 1:**
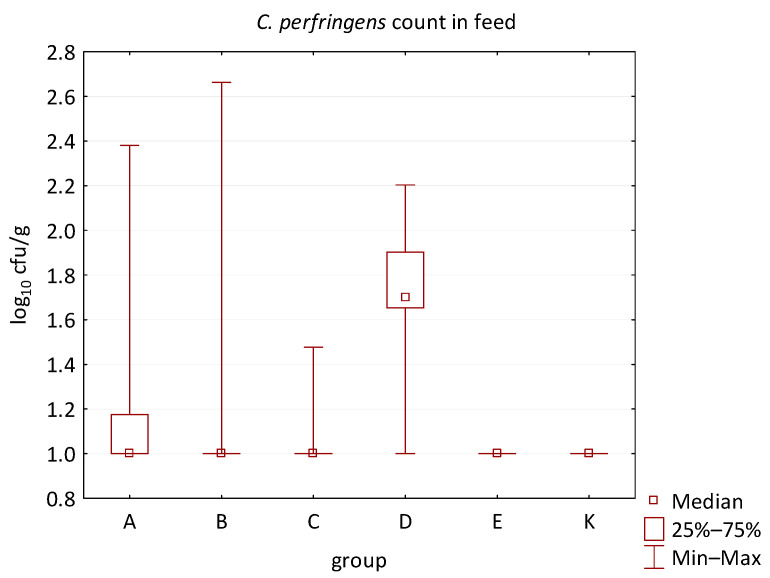
The descriptive statistics (min, max, median, 25%, and 75% percentiles) for the *C. perfringens* count in all feeds.

**Figure 2:**
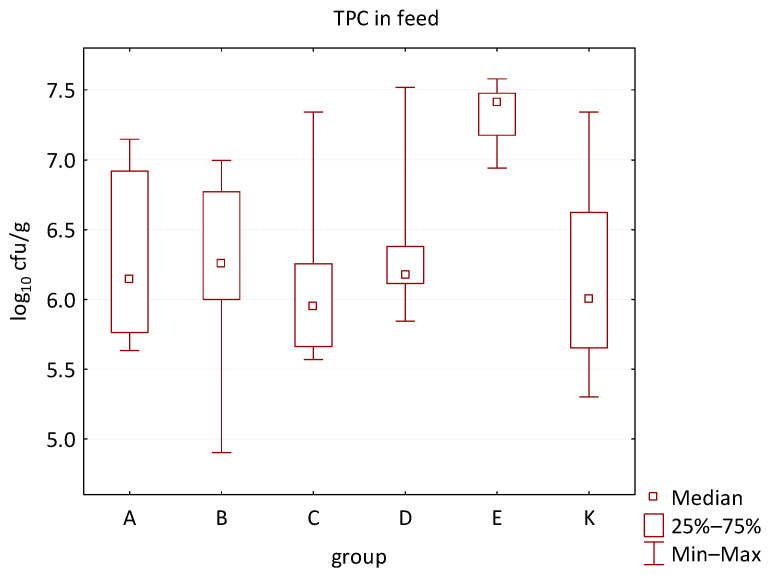
The descriptive statistics (min, max, median, 25%, and 75% percentiles) for the TPC in all feeds.

**Figure 3:**
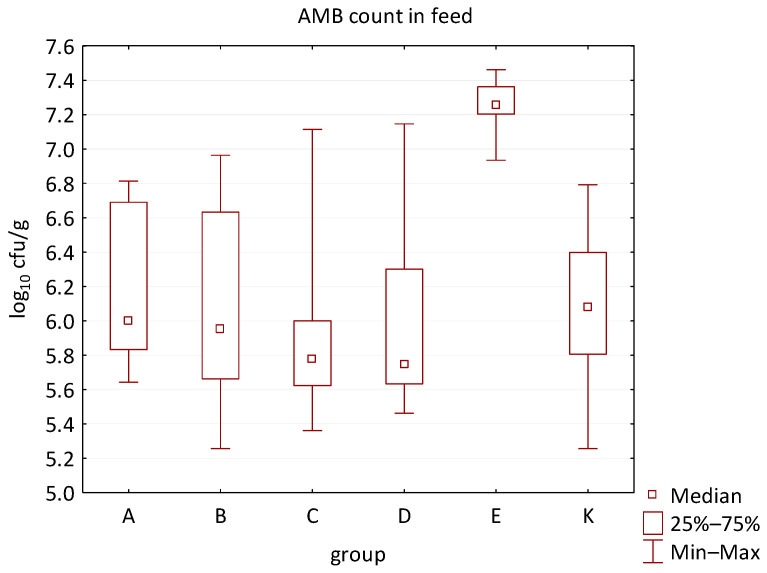
The descriptive statistics (min, max, median, 25%, and 75% percentiles) for the AMB count in all feeds.

**Figure 4:**
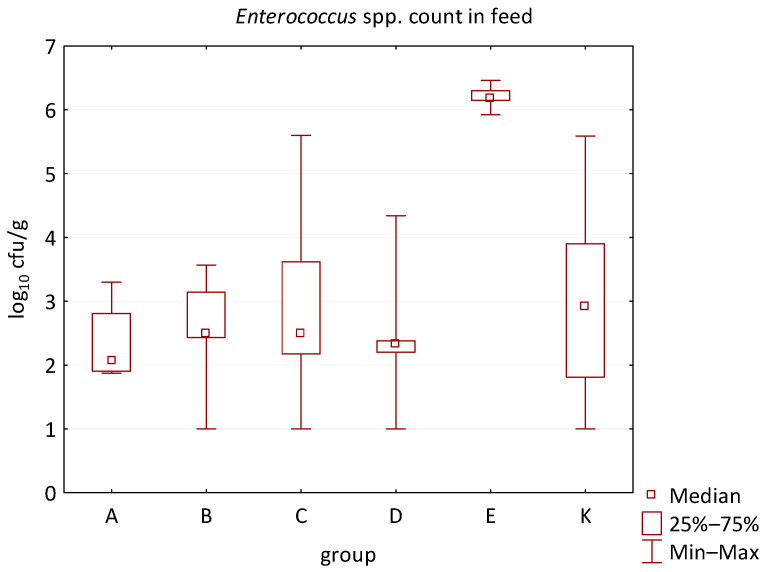
The descriptive statistics (min, max, median, 25%, and 75% percentiles) for the *Enterococcus* spp. count in all feeds.

**Figure 5:**
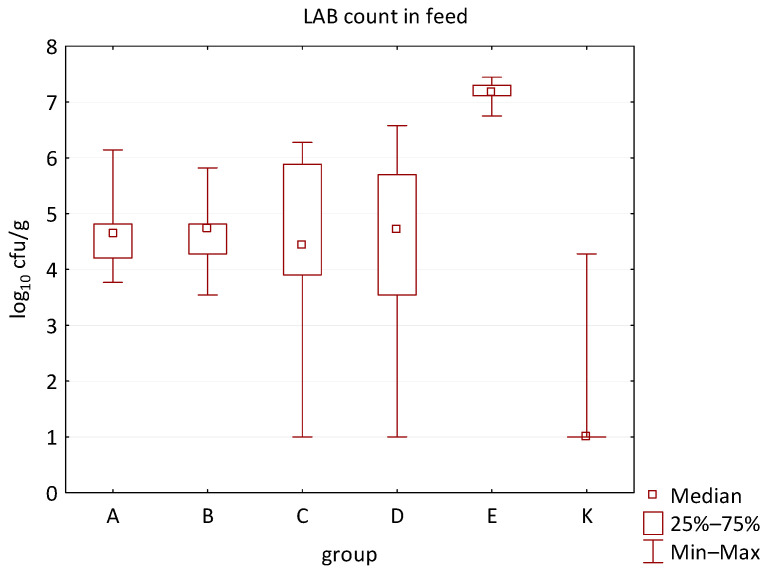
The descriptive statistics (min, max, median, 25%, and 75% percentiles) for the LAB count in all feeds.

The differences between individual groups were recorded through quantitative analyses of all feed samples (Figure 1, Figure 2, Figure 3, Figure 4 and Figure 5). Statistically significant differences were found in the count of *C. perfringens* (*p* = 0.0013), AMB (*p* = 0.0006), TPC (*p* = 0.0019), *Enterococcus* spp. (*p* = 0.0003), and LAB (*p* = 0.0000). The count of *C. perfringens* was significantly higher in group D compared to group E (*p* = 0.0486) and group K (*p* = 0.0486). The count of AMB was higher in group E compared to groups A (*p* = 0.0296), B (*p* = 0.0079), C (*p* = 0.0019), D (*p* = 0.0020), and K (*p* = 0.0083). A higher count of TPC was observed in group E compared to groups A (*p* = 0.0213), C (*p* = 0.0030), and K (*p* = 0.0043). In group E, a higher count of *Enterococcus* spp. was evident compared to groups A (*p* = 0.0007), B (*p* = 0.0081), C (*p* = 0.0224), D (*p* = 0.0005), and K (*p* = 0.0112). Similarly, a significantly higher count of LAB was found in group E compared to groups A (*p* = 0.0296), B (*p* = 0.0430), C (*p* = 0.0207), D (*p* = 0.0143), and K (*p* = 0.0000). The comparison of all supplemented feeds with the negative control feed also identified statistically significant differences. Significantly higher counts compared to group K were found for *C. perfringens* in group D (*p* = 0.0018), AMB in group E (*p* = 0.0004), TPC in group E (*p* = 0.0011), *Enterococcus* spp. in group E (*p* = 0.0004), and LAB in groups A (*p* = 0.0006), B (*p* = 0.0005), C (*p* = 0.0066), D (*p* = 0.0066), and E (*p* = 0.0002). In feed for sows, the LAB count was significantly higher in group E than in group K (*p* = 0.0114). Comparison of feed from individual groups with the negative control feed showed no differences.

The difference between the type of feed additive and the count of *Clostridium* spp. (*p* = 0.0636) and *C. perfringens* (*p* = 0.0593) in group D, as well as the count of *Clostridium* spp. (*p* = 0.0722) in group A, was close to the limit of significance. Moreover, the LAB count in groups A, B, D, and E (*p* = 0.0765) and the count of *Enterococcus* spp. in group E (*p* = 0.0765) were close to significance as well. On the borderline of statistical significance was the occurrence of *C. perfringens* in groups A, B, C, and D. In feed intended for piglets, the LAB count was significantly higher in group E than in group K (*p* = 0.0197). No differences were identified when comparing the supplemented feeds and the negative control feed. Close to the level of significance (*p* = 0.0636) was the count of LAB in groups A, B, and E. In feed for fatteners, the LAB count was also significantly higher in group E than in group K (*p* = 0.0197). No differences were identified when comparing the supplemented feeds and the negative control feed. Close to the level of significance (*p* = 0.0636) was the count of LAB in groups A, B, and E. The Pearson chi-square test found a correlation between the occurrence of *C. perfringens* and the type of feed additive (*p* = 0.0274), but no statistically significant differences were found in single comparisons between the supplemented feeds and the negative control feed. In other microbiological parameters, no differences were shown in feed for sows, piglets, and fatteners between all groups of the trial.

### 3.2. Animal Raw Materials

**Figure 6:**
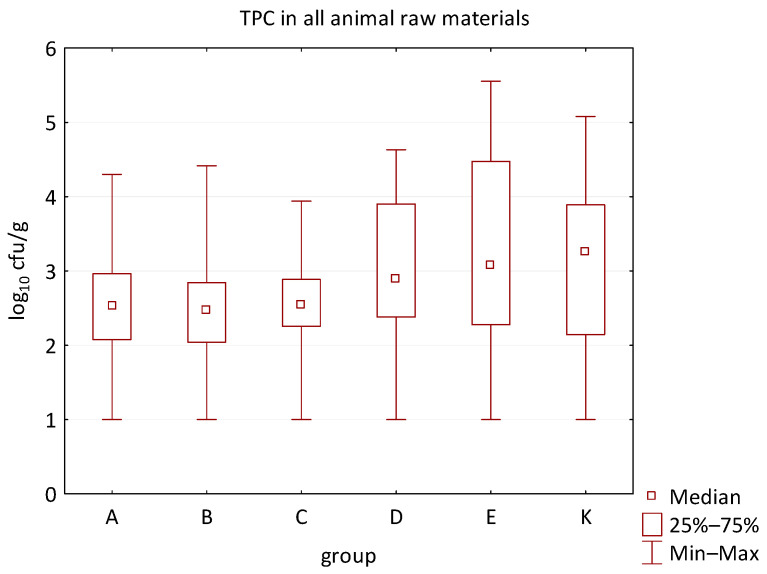
The descriptive statistics (min, max, median, 25%, and 75% percentiles) for the TPC in all animal raw materials.

**Figure 7:**
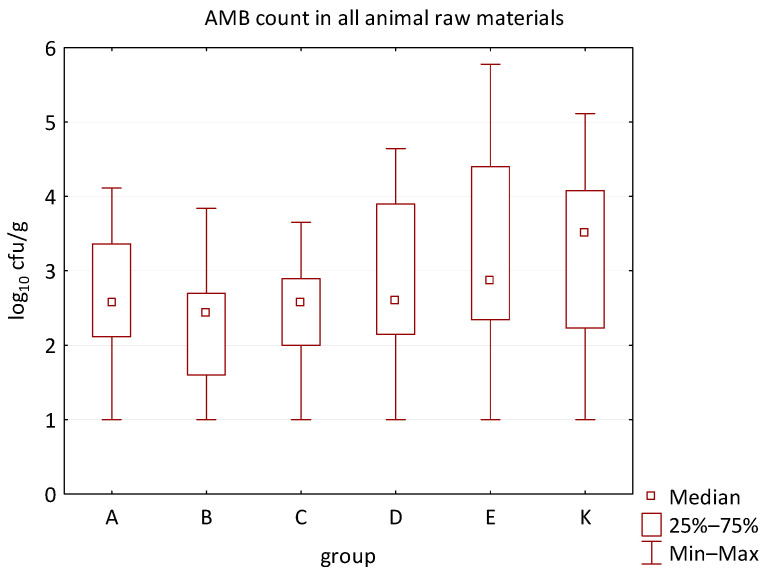
The descriptive statistics (min, max, median, 25%, and 75% percentiles) for the AMB count in all animal raw materials.

**Figure 8:**
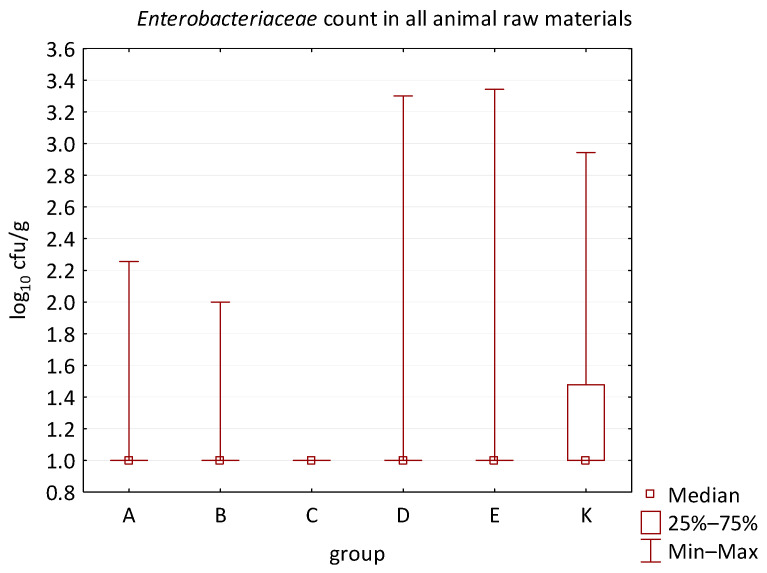
The descriptive statistics (min, max, median, 25%, and 75% percentiles) for the Enterobacteriaceae count in all animal raw materials.

**Figure 9:**
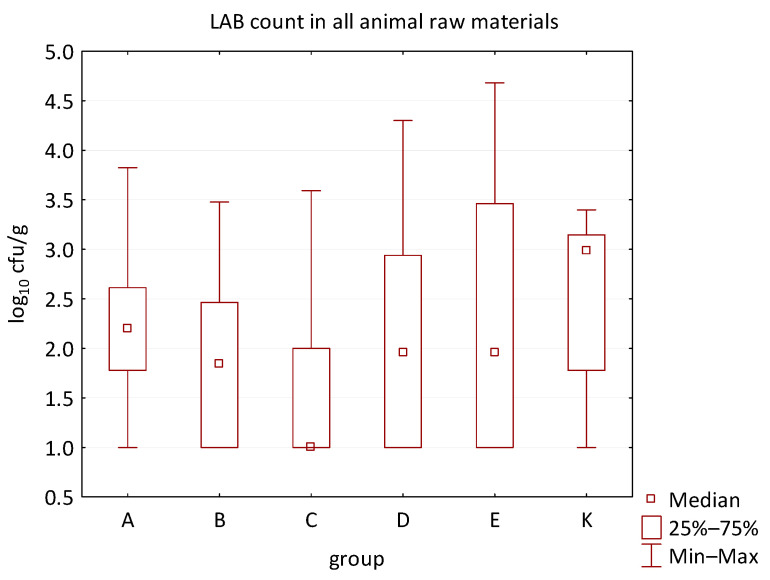
The descriptive statistics (min, max, median, 25%, and 75% percentiles) for the LAB count in all animal raw materials.

No difference was identified between the feed additives supplementation and the occurrence of zoonotic agents in edible raw materials derived from all animals (Table 5). However, the microbial quantitative data identified statistically significant correlations between the microbial count of all raw materials and the type of supplemented feed additive (Figure 6, Figure 7, Figure 8 and Figure 9). Differences were identified in the count of LAB (*p* = 0.0014) between groups C and K (*p* = 0.0008). In addition, there were significant differences in the count of Enterobacteriaceae (*p* = 0.0324) and AMB (*p* = 0.0161). However, the multiple comparison test did not confirm these differences, and only the count of AMB between feed B and K was close to the limit of significance (*p* = 0.0543). Moreover, the comparison of experimental groups A–E with the negative control raw materials demonstrated differences in the count of AMB, TPC, Enterobacteriaceae, and LAB. The Enterobacteriaceae count differed significantly between groups A and K (*p* = 0.0450) as well as C and K (*p* = 0.0026). The AMB count of group A (*p* = 0.0339), B (*p* = 0.0067), and C (*p* = 0.0101) was significantly lower than in the negative control group K. Additionally, the TPC differed between B and K groups (*p* = 0.0385), and the count of LAB was significantly different between groups B and K (*p* = 0.0020) as well as C and K (*p* = 0.0000).

**Figure 10:**
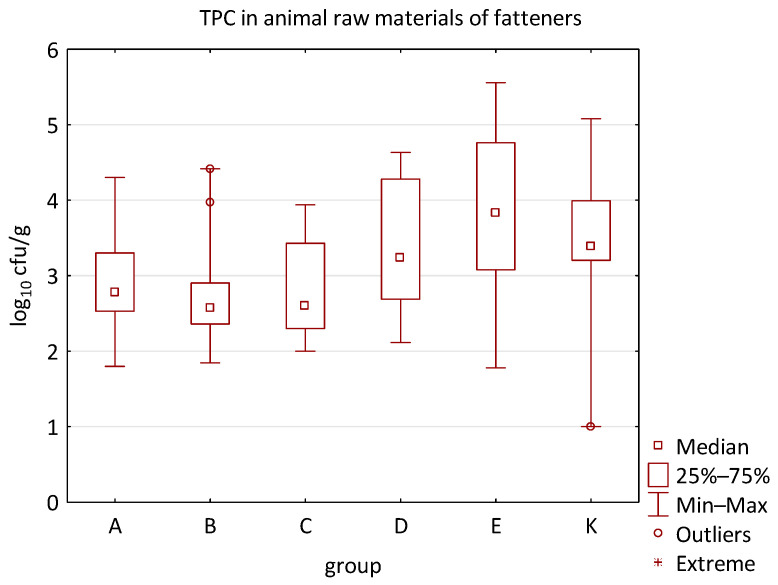
The descriptive statistics (min, max, median, 25%, and 75% percentiles) for the TPC in animal raw materials of fatteners.

**Figure 11:**
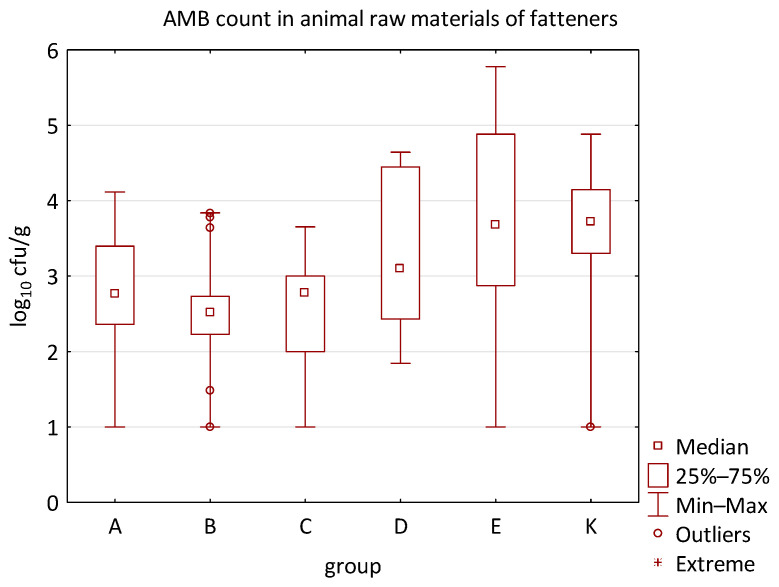
The descriptive statistics (min, max, median, 25%, and 75% percentiles) for the AMB count in animal raw materials of fatteners.

**Figure 12:**
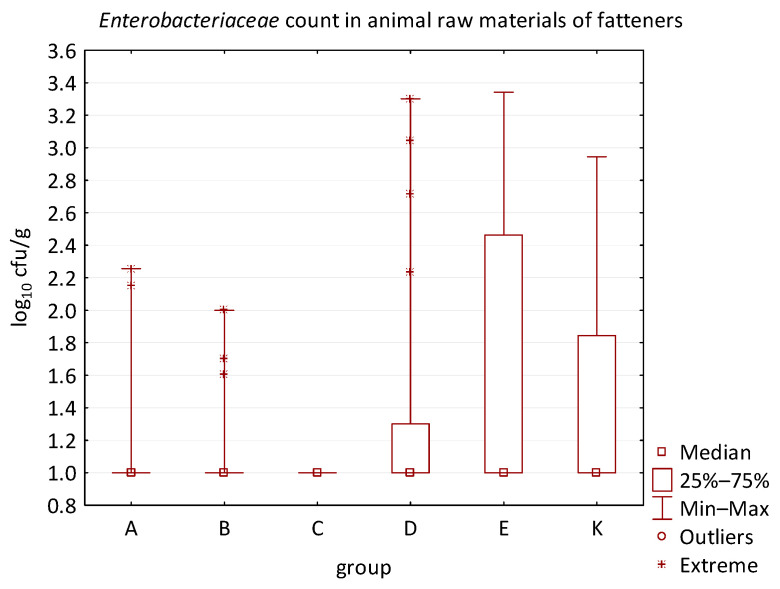
The descriptive statistics (min, max, median, 25%, and 75% percentiles) for the Enterobacteriaceae count in animal raw materials of fatteners.

**Figure 13:**
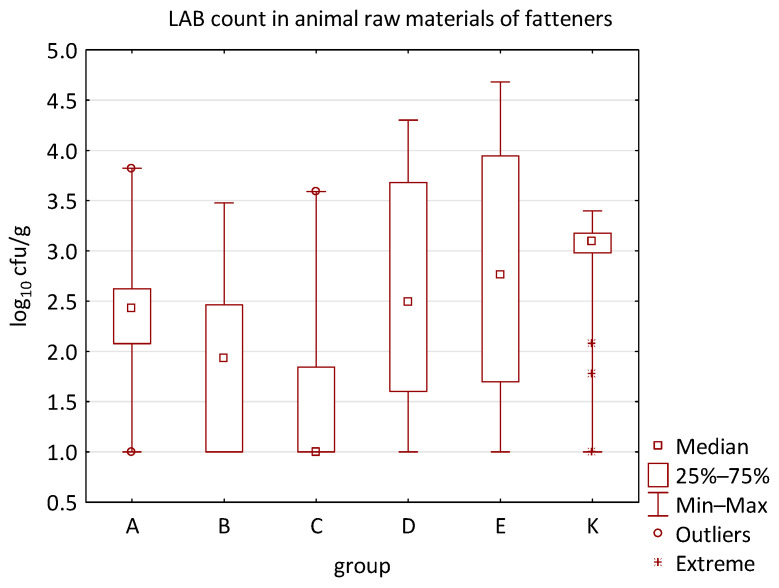
The descriptive statistics (min, max, median, 25%, and 75% percentiles) for the LAB count in animal raw materials of fatteners.

The microbial load of edible raw materials from piglets did not show any significant differences between experimental groups in both qualitative and quantitative indicators. Similarly, the occurrence of zoonotic agents in raw materials of fattening pigs did not differ between groups in the experiment. However, analysis of quantitative data found a difference between the microbial load of edible raw materials of fattening pigs and the type of feed additive (Figure 10, Figure 11, Figure 12 and Figure 13). A statistically significant difference was found in LAB count between groups C and E (*p* = 0.0104), B and K (*p* = 0.0240), and C and K (*p* = 0.0002) where LAB count was significantly higher in groups E and K. The AMB count and TPC in individual groups also differed significantly, with *p*-values of *p* = 0.0001 and *p* = 0.0004, respectively. The AMB count differed between groups B and E (*p* = 0.0090), C and E (*p* = 0.0109), B and K (*p* = 0.0028), and C and K (*p* = 0.0035). The difference in TPC was demonstrated between groups B and E (*p* = 0.0120), C and E (*p* = 0.0252), and B and K (*p* = 0.0256). In addition, the Kruskal–Wallis test demonstrated differences in Enterobacteriaceae count (*p* = 0.0215), but it was not confirmed by the multiple comparisons test.

Furthermore, the microbial burden of raw materials in groups A-E demonstrated a correlation when compared to the negative control group. Differences were found in the Enterobacteriaceae count between groups A and K (*p* = 0.0391) and C and K (*p* = 0.0019); in the AMB count between groups A and K (*p* = 0.0021), B and K (*p* = 0.0002), and C and K (*p* = 0.0001); in the TPC between groups A and K (*p* = 0.0095), B and K (*p* = 0.0014), C and K (*p* = 0.0040); and in the LAB count between groups A and K (*p* = 0.0053), B and K (*p* = 0.0004), and C and K (*p* = 0.00005). The Enterobacteriaceae count in the raw materials of group K was significantly higher compared to groups A and C. Similarly, the AMB count was higher in group K compared to groups A, B, and C. Moreover, the LAB count in groups A, B, and C was significantly lower than in the negative control raw materials. Other quantitative results, such as the count of *E. coli*, *C. perfringens*, *Clostridium* spp., *B. cereus*, HS, CoPS, *Enterococcus* spp., fungi, or yeast, did not show statistically significant differences in individual groups of the trial. 

Differences were observed in synbiotic preparations with a higher number of probiotic strains. This was observed in the raw materials of all animals at the Enterobacteriaceae count of group A (*p* = 0.0450) and C (*p* = 0.0026); AMB count of group A (*p* = 0.0339), B (*p* = 0.0067), and C (*p* = 0.0101); TPC of group B (*p* = 0.0385); LAB count in group B (*p* = 0.0020) and C (*p* = 0.00007); as well as in the raw materials of fatteners at the Enterobacteriaceae count of group A (*p* = 0.0391) and C (*p* = 0.0019); AMB count of group A (*p* = 0.0021), B (*p* = 0.0002), and C (*p* = 0.0001); TPC of group A (*p* = 0.0095), B (*p* = 0.0014), and C (*p* = 0.0040); or LAB count of group A (*p* = 0.0053), B (*p* = 0.0004), and C (*p* = 0.0000).

**Figure 14:**
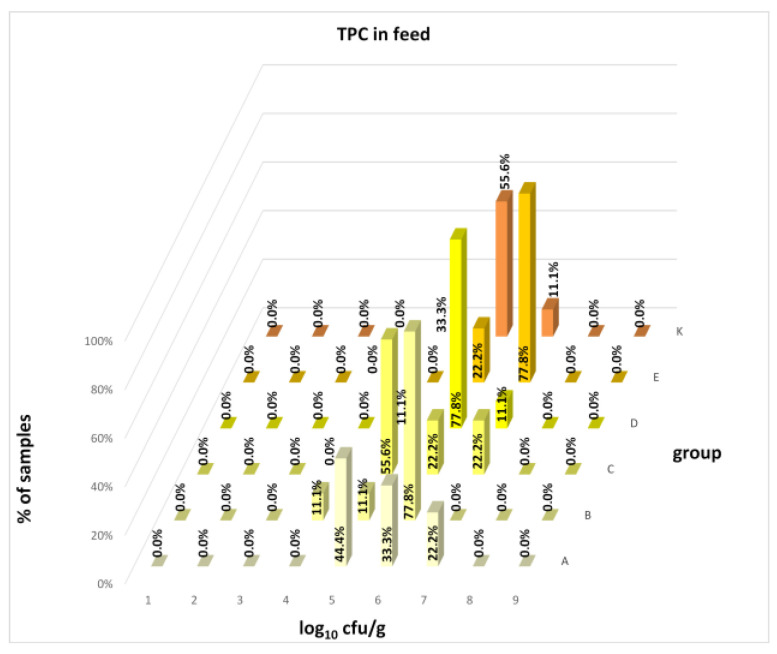
Distribution of TPC in feeds: the vertical axis (Y) represents the percentage frequency of observations; the horizontal axis (X) represents the count of microorganisms on a logarithmic scale; the third axis (Z) represents the experimental group.

**Figure 15:**
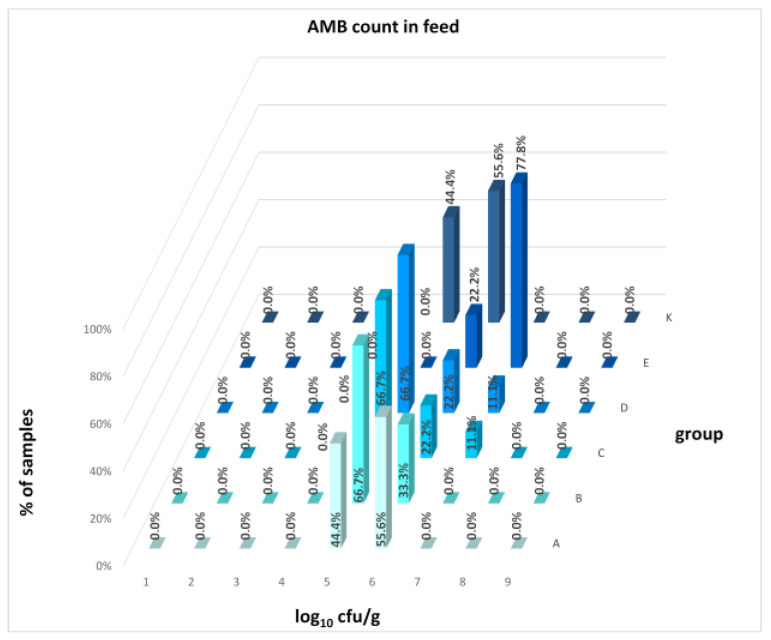
Distribution of AMB count in feeds.

**Figure 16:**
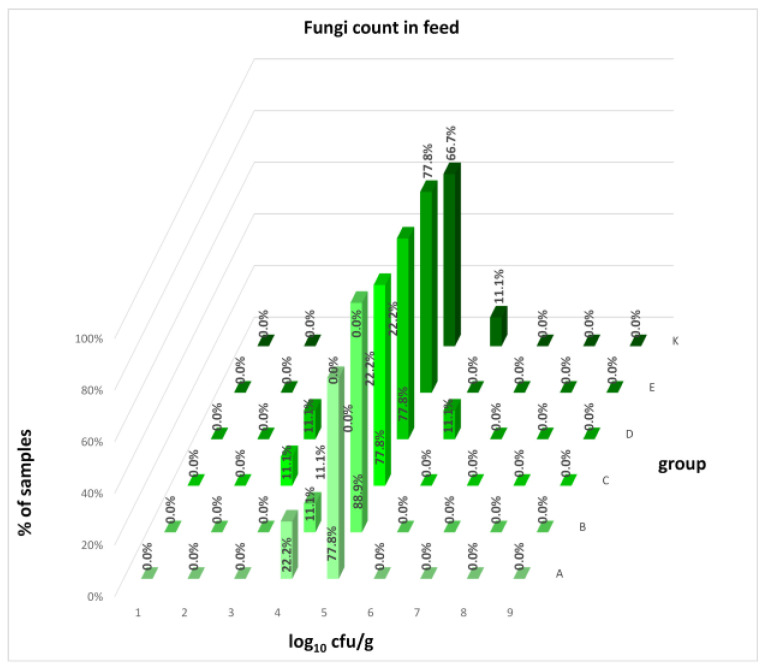
Distribution of fungi count in feeds.

**Figure 17:**
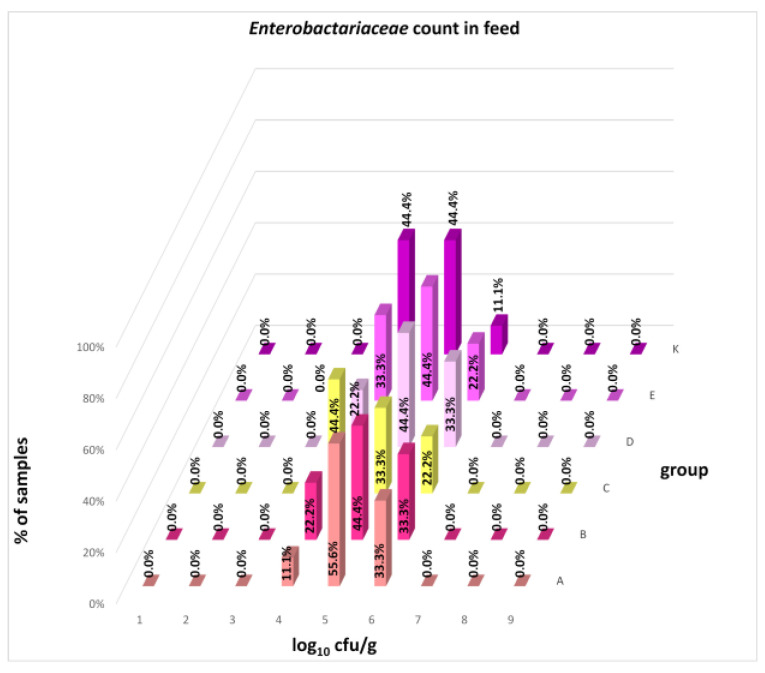
Distribution of Enterobacteriaceae count in feeds.

**Figure 18:**
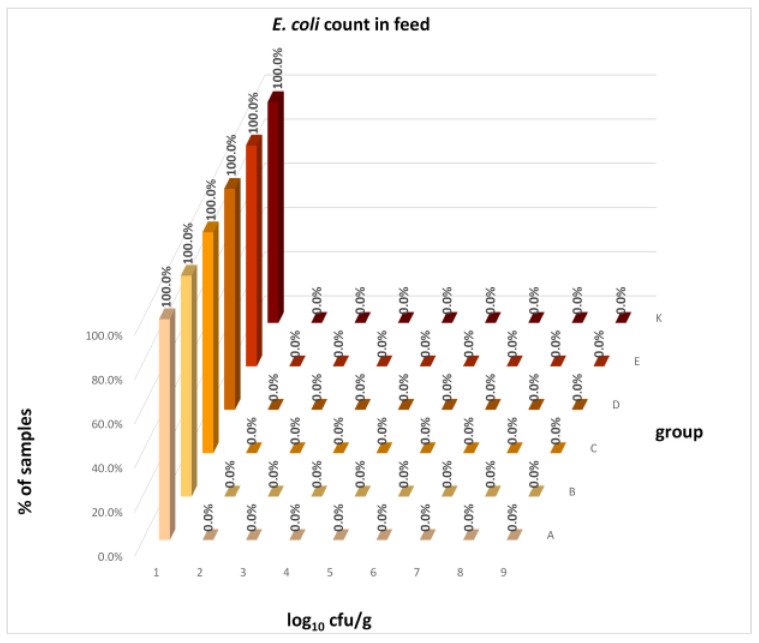
Distribution of *E. coli* count in feeds.

**Figure 19:**
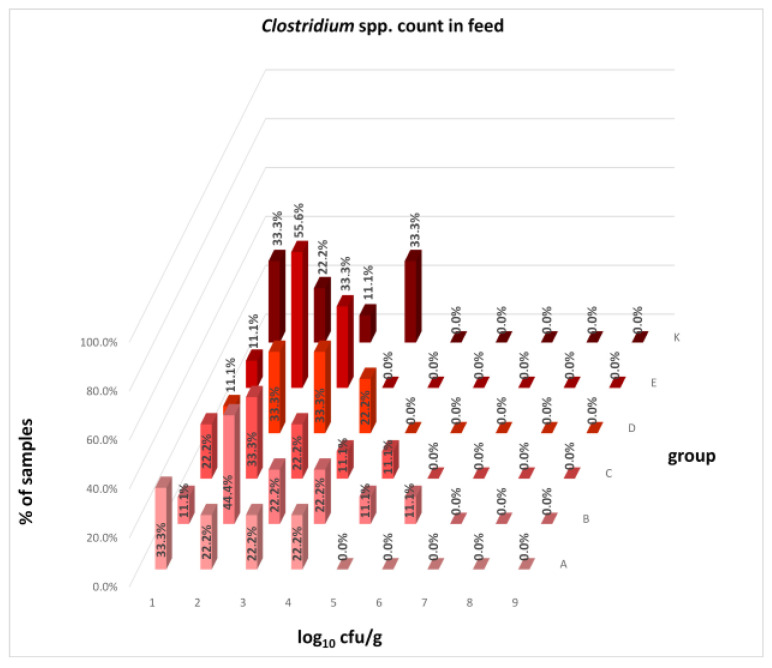
Distribution of *Clostridium* spp. count in feeds.

**Figure 20:**
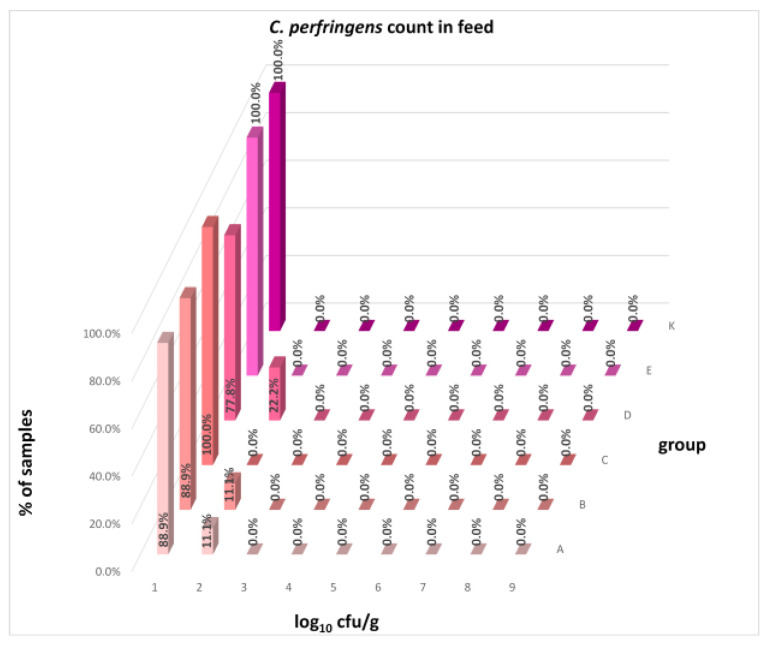
Distribution of *C. perfringens* count in feeds.

**Figure 21:**
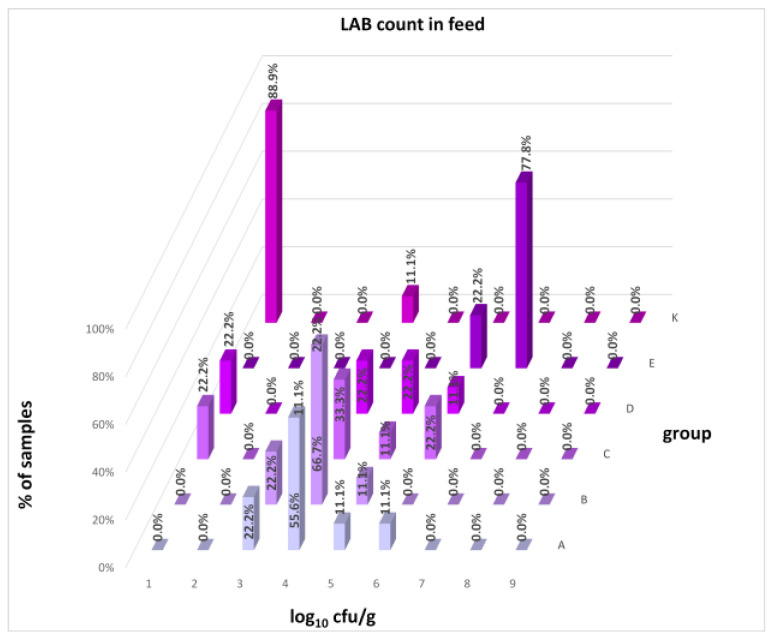
Distribution of LAB count in feeds.

**Figure 22:**
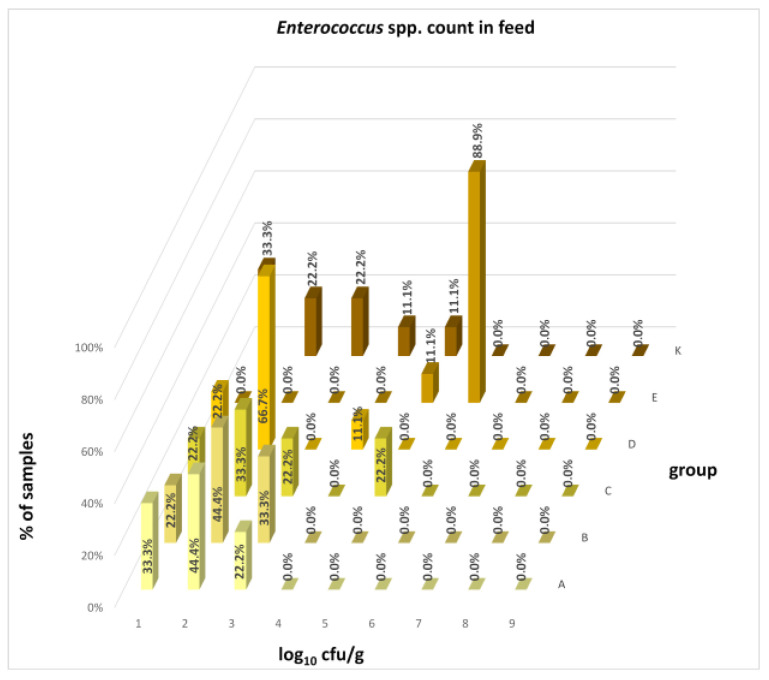
Distribution of *Enterococcus* spp. count in feeds.

**Figure 23:**
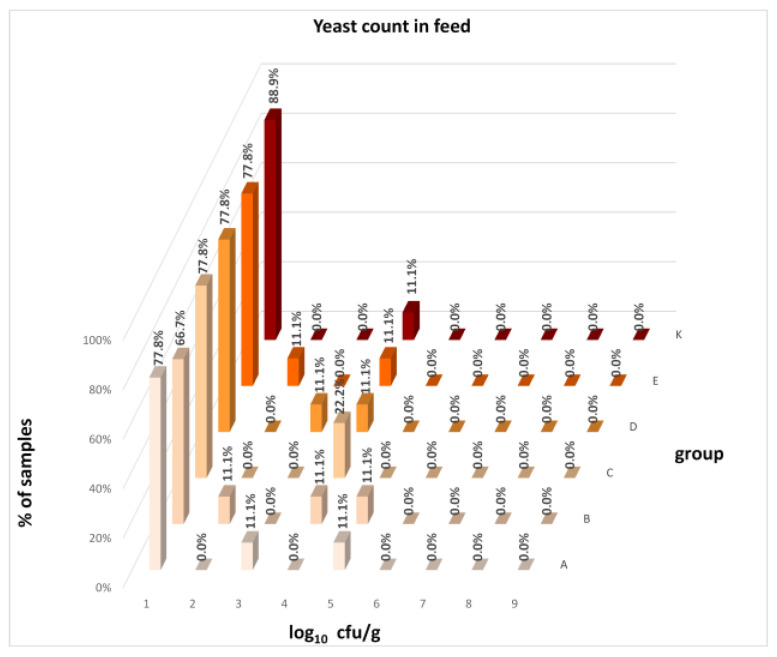
Distribution of yeast count in feeds.

**Figure 24:**
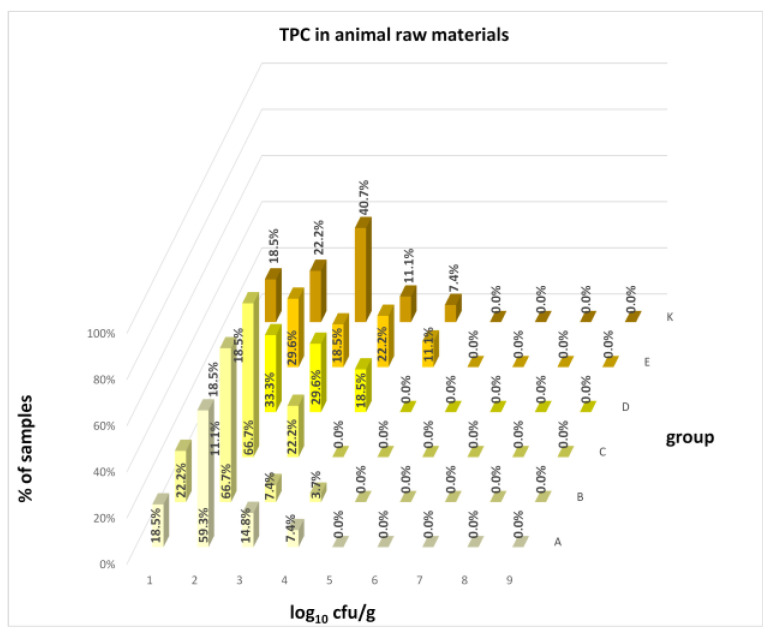
Distribution of TPC in animal raw materials.

**Figure 25:**
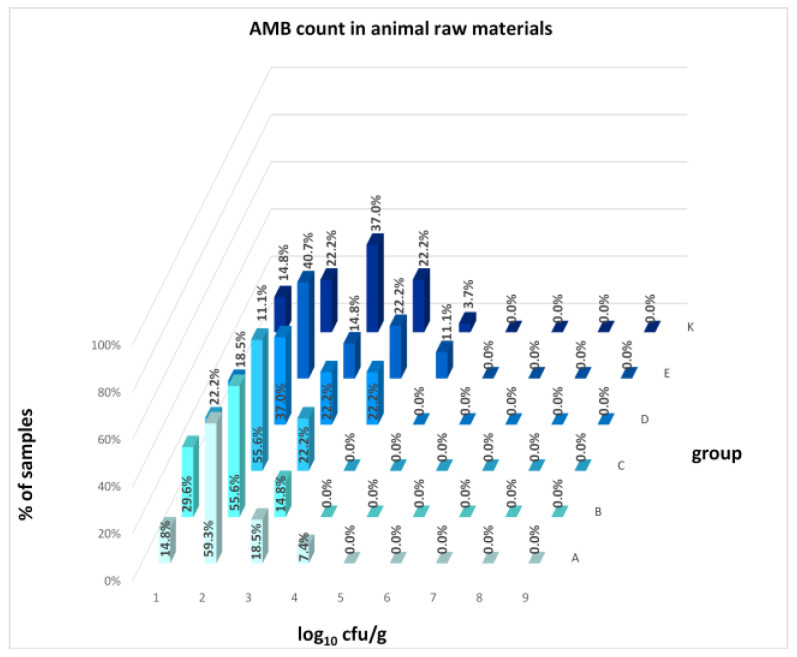
Distribution of AMB count in animal raw materials.

**Figure 26:**
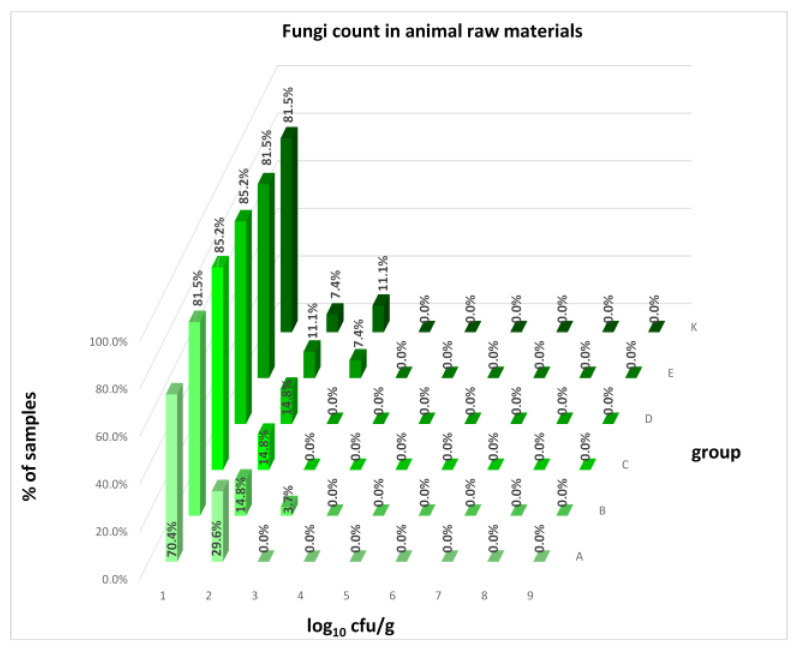
Distribution of fungi count in animal raw materials.

**Figure 27:**
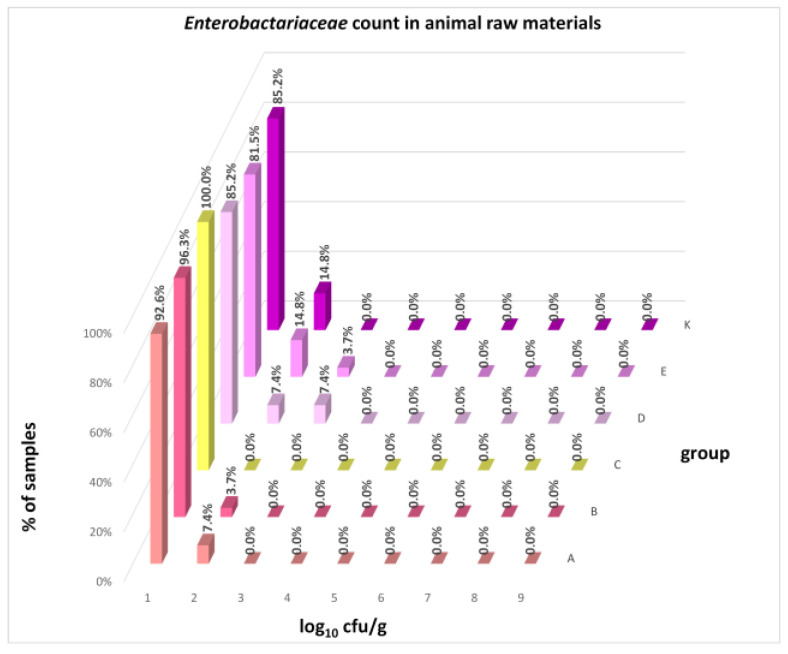
Distribution of *Enterobacteriaceae* count in animal raw materials.

**Figure 28:**
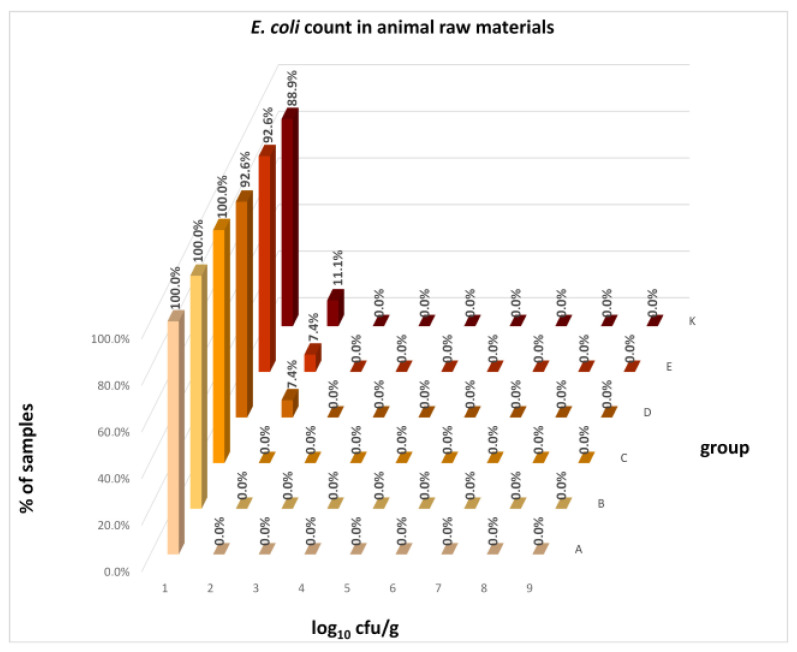
Distribution of *E. coli* count in animal raw materials.

**Figure 29:**
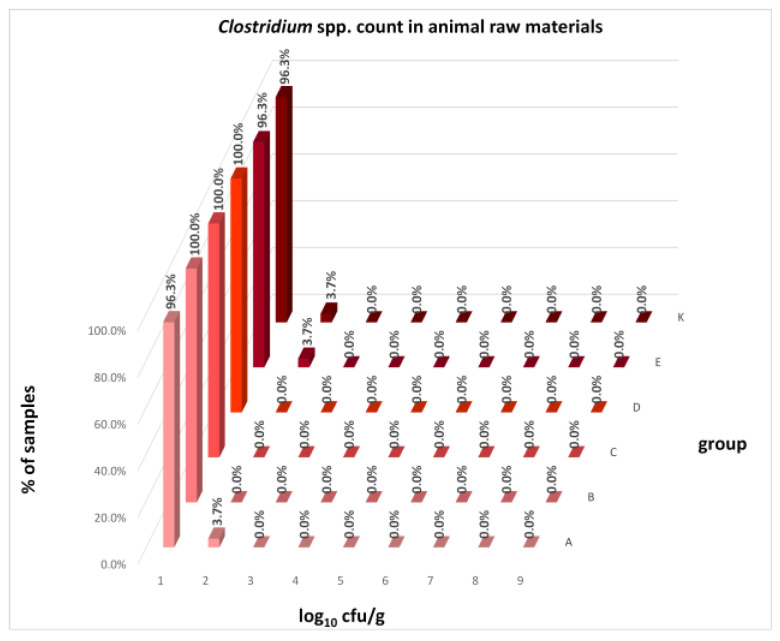
Distribution of *Clostridium* spp. count in animal raw materials.

**Figure 30:**
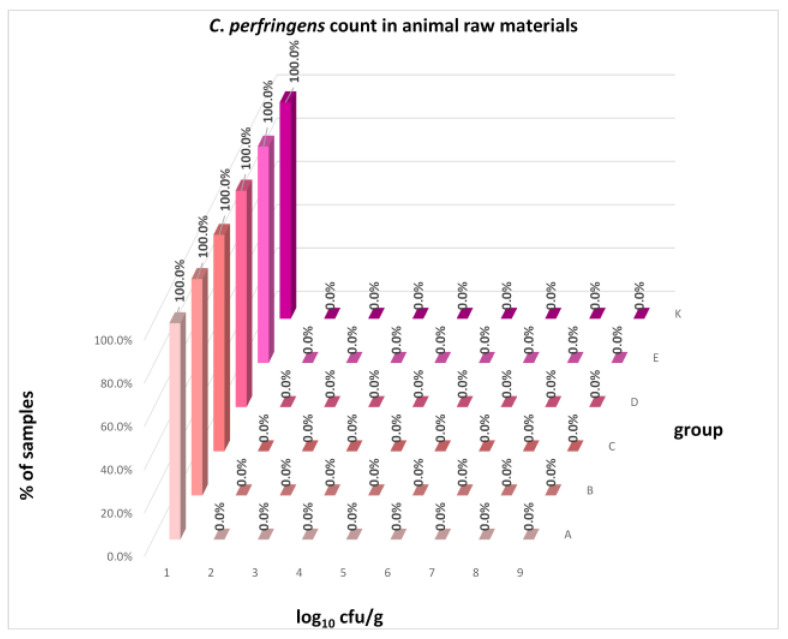
Distribution of *C. perfringens* count in animal raw materials.

**Figure 31:**
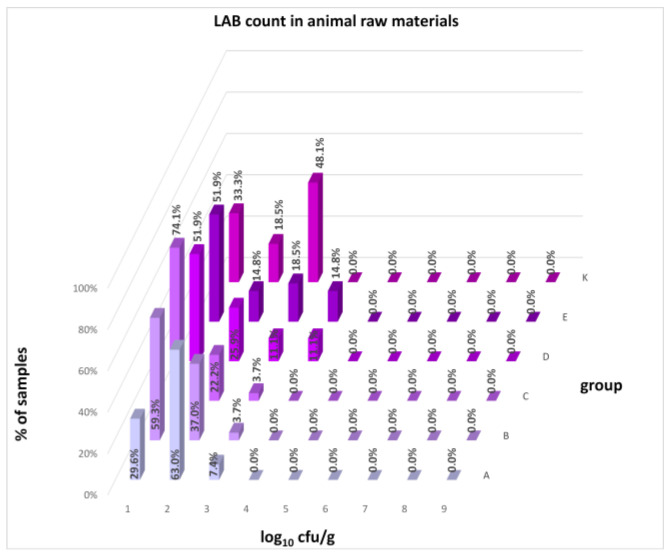
Distribution of LAB count in animal raw materials.

**Figure 32:**
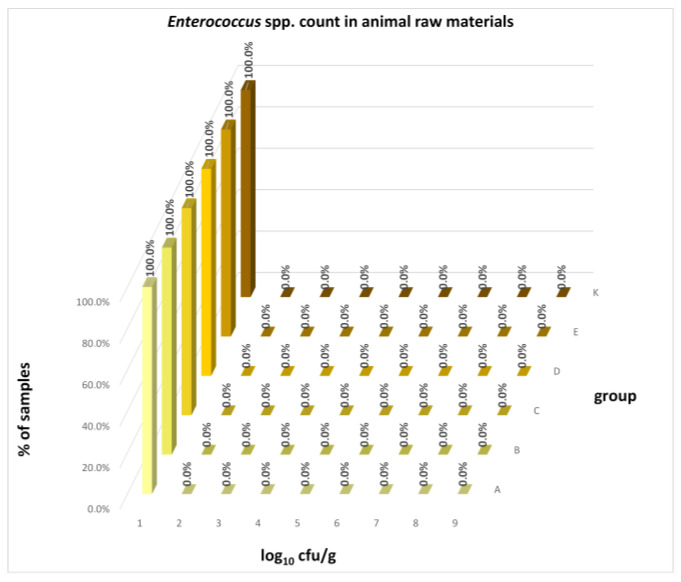
Distribution of *Enterococcus* spp. count in animal raw materials.

**Figure 33:**
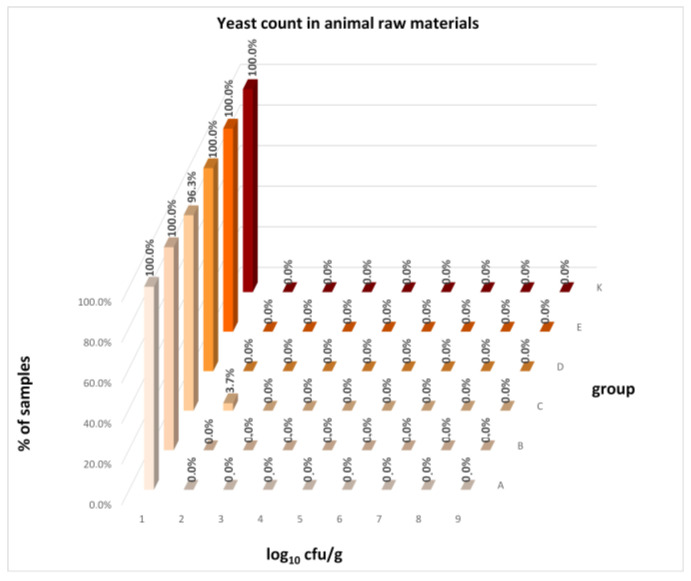
Distribution of yeast count in animal raw materials.

The distribution of individual microorganism levels in feed, including all experimental groups, is shown in Figure 14, Figure 15, Figure 16, Figure 17, Figure 18, Figure 19, Figure 20, Figure 21, Figure 22 and Figure 23, and Figure 24, Figure 25, Figure 26, Figure 27, Figure 28, Figure 29, Figure 30, Figure 31, Figure 32 and Figure 33 demonstrate the distribution of individual microorganism levels in animal raw materials. TPC and AMB counts fall between 5 and 7 log_10_ cfu/g in most samples of feeds, in contrast to those of animal raw materials, where these indicator levels commonly range from 1 to 3 log_10_ cfu/g. Most samples of feed were contaminated with fungi at levels of 4–5 log_10_ cfu/g, and animal raw materials were contaminated at levels of 1–2 log_10_ cfu/g. Pathogen indicators such as the Enterobacteriaceae family, *E. coli*, *C. perfringens*, and *Clostridium* spp. were extremely low in animal raw materials, similar to the levels of *E. coli* and *C. perfringens* found in feed samples, with nearly 90% to 100% of samples at a 1 log_10_ cfu/g count. In the Enterobacteriaceae family, the count of most feed samples ranged from 4 to 6 log_10_ cfu/g, and *Clostridium* spp. ranged from 1 to 4 log_10_ cfu/g. Most samples of animal raw materials showed a LAB count from 1 to 2 log_10_ cfu/g, whereas feed samples demonstrated levels of 3 to 5 log_10_ cfu/g. *Enterococcus* spp. and yeast counts were extremely low in animal raw materials, with nearly 100% of samples at a 1 log_10_ cfu/g count. In contrast, *Enterococcus* spp. counts in feed ranged from 1 to 3 log_10_ cfu/g, and yeast counts were 1 log_10_ in 67–89% of samples.

## 4. Discussion

The impact of synbiotics on human and animal health is indisputable, and new modes of action of these additives are still being discovered [50,51]. So far, research on the microbial status of feed supplemented with synbiotics has not been carried out, and the effect on the hygienic quality of meat and offal derived from animals supplemented with synbiotics is still emerging [17]. The first objective of our study was to test the influence of newly created synbiotic preparations on the microbial status of feed for swine of different ages in comparison with commercially available probiotics and a negative control. This experiment pointed out that the prevalence of *C. perfringens* was decreased by synbiotics A, B, C, and probiotic D in feed for sows, as well as synbiotic C and probiotic D in feed for fatteners. These results are particularly promising in the context of the growing problem of antimicrobial resistance. Recent literature indicates that the use of synbiotics as an ecological alternative to traditional growth promoters can allow for effective control of pathogens such as *C. perfringens* through the production of short-chain fatty acids (butyrate, acetate, propionate) [52,53]. This study confirms that modulation of the feed microbiome using preparations A, B, and C is an important element of the ‘One Health’ strategy. To the best of our knowledge, there is no data regarding the influence of synbiotics on the hygienic status of feed. Therefore, this finding should be strengthened and confirmed in future studies with a different approach that can provide more definitive results. Analysis of total feed samples revealed that the prevalence and count of *C. perfringens* were higher if probiotic D was added, and the supplementation of probiotic E reduced the load of feed by the anaerobe, simultaneously increasing their total microbial load. However, this had no effect on the European Food Safety Authority Panel on Additives and Products or Substances used in Animal Feed opinions, confirming that both feed additives have the potential to improve the performance of pigs for fattening [54,55]. The effect of newly created synbiotics on the microbial status of edible raw materials of pig origin supplemented with these synbiotics was the second goal of our study. The design of the animal trial aimed to indirectly check whether synbiotics may influence the crossing barrier of the digestive tract by intestinal microflora and, consequently, microbial contamination of pig carcasses. The study evidenced that the prevalence of zoonotic agents in edible raw materials of piglets and fatteners was not changed by the synbiotics or probiotics tested. However, in contrast to the microbial qualitative (pathogen) indicators, we did observe changes in the microbial load of raw materials originating from animals supplemented with synbiotics. These changes were noted in analyses of both total raw materials and raw materials of fatteners. Synbiotics A, B, and C significantly reduced the count of AMB, TPC, and LAB, and Enterobacteriaceae family contamination was decreased by synbiotics A and C. The tested raw materials revealed a lower microbial load by aerobic mesophilic bacteria (AMB); aerobic or microaerophilic microorganisms including mold, yeast, and bacteria (TPC); and the Enterobacteriaceae family, considered general indicators of food hygiene. The significantly lower LAB count in raw materials originating from synbiotic-supplemented pigs is especially worth noting, particularly as the count of LAB in feed supplemented with synbiotics was significantly higher than the negative control. Consequently, the count of LAB was higher both in pigs’ fecal microbiota of groups A, B, and C, as estimated by other researchers analyzing pig intestinal content in the same animal trial [56]. A higher count of LAB in both feed and fecal microbiota, with a lower count of LAB in raw materials of these animals, confirms the positive role of tested synbiotics on the hygienic quality of pork. According to our best knowledge, this is the first report on the decrease in general hygienic indicators in edible pork raw materials due to supplementation with synbiotics. So far, the literature data found synbiotics decreasing *Salmonella* contamination of carcasses in pigs, which is in compliance with the drop in Enterobacteriaceae contamination in our study [57]. Other experiments demonstrated that dietary supplementation with synbiotics reduced *Salmonella* and *C. jejuni* load post-harvest in broiler chicken carcasses [14,17]. A study in broiler birds showed that supplementation of a synbiotic containing *L. reuteri*, *E. faecium*, *B. animalis*, *P. acidilactici*, and a fructooligosaccharide from the day of hatch decreased *S.* Enteritidis load in the cecal tonsils, reducing carcass contamination by 1.5 log units/mL of rinsate compared to the control group [58]. Interestingly, the animal study compiled with the challenge of a pathogen reversed the above results. An American study found that synbiotic supplementation did not limit liver *C. jejuni* load in chicken broilers challenged with *C. jejuni* compared to the control group [59]. Likewise, significantly greater colonization of internal organs (liver, spleen, kidneys, palatine tonsils, mandibular lymph nodes, jejunal lymph nodes, muscle samples from the forelimb, hind limb, and diaphragm) with *S.* Typhimurium was observed in pigs treated with *E. faecium* probiotic relative to controls [60]. In contrast to studies where synbiotics failed to limit colonization of internal organs under severe pathogen challenge, our results suggest that under standard pig production conditions, synbiotics effectively prevent microbial translocation [58,59,60]. This may be because the tested preparations acted prophylactically, building a stable microbiota from the sow’s lactation period through fattening, which is a more effective strategy than intervention at the time of infection. Further light on the activity of tested synbiotics in pigs was shed by two other research teams analyzing other aspects of the same animal trial. A team from Lodz University of Technology showed that newly developed synbiotics significantly increased the beneficial bacteria population (*Lactobacillus* spp., *Bifidobacterium* spp., *Bacteroides* spp.) and decreased the count of potential pathogens (*Clostridium* spp., *Enterococcus* spp., Enterobacteriaceae family, *E. coli*) in the feces of piglets from nursing to fattening [56]. Simultaneously, the second team from the Department of Swine Diseases in NVRI demonstrated enhanced immune activity with a significant increase in serum immunoglobulin concentration in sows and growing pigs by B and C synbiotics [15]. The results of studies of various aspects in this one animal study by three independent teams are cohesive with each other and indicate combined effects of synbiotics in pigs. The lower microbial burden of pork raw materials demonstrated in our study was due to the modulation of intestinal microbiota, restoring the integrity of the protective intestinal mucosa, and positive modulation of the immune response by the components of synbiotics shown by two other studies. Interpretation of the above results shows that synbiotics reduce the potential pathogen load of the intestine, resulting in a decrease in the microbial load of pig raw materials. The above reveals synbiotic supplementation in animal production as an effective approach to decreasing the microbial load of pig carcasses. The association between pigs with high cecal *Salmonella* loads and carcass contamination has already been noted. First, authors showed that the reduction in *Salmonella* loads in the guts of slaughtered pigs results in fewer contaminated carcasses, consequently helping to minimize the risk of human infection due to the consumption of contaminated pork [57]. A second study demonstrated synbiotic supplementation decreasing the *Salmonella* load in both cecal content and carcass rinsate of broiler birds, suggesting that synbiotics not only efficiently colonized the intestine but also secreted antibacterial substances in the gut lumen to decrease the *S.* Enteritidis load in the carcass [58]. This property of synbiotics is of particular value as good hygiene quality of edible animal raw materials is strongly desirable in the agri-food sector, and their initial microbial load is essential for the safety of food of animal origin and its shelf life. The analogous activity to protection and restoration of intestinal permeability in vitro and in vivo has been previously demonstrated in probiotics [61,62,63,64]. Trials in animal models have shown that probiotics improved intestinal barrier function due to a reduction in the permeability of the intestinal epithelium. Translocation of intestinal microbes out of intestinal sites and into sites such as the liver, spleen, and mesenteric lymph nodes was decreased in mice with induced colitis and pre-treated with *Lactobacillus* probiotics [65]. Translocation of enterotoxigenic *E. coli* to mesenteric lymph nodes was reduced in post-weaning piglets with dietary supplementation of probiotic *Pediococcus acidilactici* compared with the control group after an enterotoxigenic *E. coli* challenge [66]. The risk difference analysis revealed that intestinal permeability was improved by up to 48% in the probiotics-supplemented group of humans compared to the negative control [61]. While previous studies have focused primarily on the elimination of specific pathogens (*Salmonella*, *C. jejuni*, *E. coli*), our results provide unique evidence that synbiotics can reduce general hygiene parameters (AMB, TPC) in edible raw materials. This ‘inverse correlation’—higher LAB counts in feces with lower LAB counts in meat—suggests that synbiotics enhance the integrity of the intestinal barrier, which is consistent with recent observations of improved tight junction integrity following microbial supplementation [67,68]. There is a growing amount of evidence that synbiotics have a more beneficial impact on animal health and contamination of carcasses compared to probiotics, including our bioassay [60,69,70]. These data are of high interest in the case of edible animal raw materials, as foodborne zoonoses continue to pose a serious threat to human health within and outside the European Union [21]. As synbiotics exert multiple benefits through various modes of action, an additional concept of our study was to compare the effectiveness of synbiotics with different numbers of strains. Higher effectiveness was associated with the supplementation of a higher number of strains in one synbiotic preparation. This was evidenced both in raw materials derived from all pigs as well as only fatteners at the count of AMB, TPC, Enterobacteriaceae, or LAB. The greater effectiveness of multi-strain preparations B and C in our study aligns with the growing scientific consensus. This effectiveness results from several factors, including interspecies synergy between different strains working together more effectively than single strains, a broader spectrum of protective activity, higher effectiveness in increasing beneficial microorganisms, decreasing potential pathogens in feces, and enhancing immune activity through higher serum immunoglobulin concentrations. However, it should be noted that although multi-strain preparations demonstrate a broader spectrum of protective activity, their effectiveness is highly strain-dependent, not solely species-dependent [15,71]. Our observations confirm that the appropriate selection of complementary strains and the prebiotic (inulin) is crucial for achieving optimal immune modulation and improving meat hygiene parameters. So far, higher effectiveness of multi-strain feed additives has been evidenced in probiotic preparations. The literature data confirmed that multi-strain probiotics are more efficient than single-strain probiotics in terms of their protective efficacy in elderly subjects and preterm infants [61,72]. An additional merit of tested synbiotics in our study is their multispecies nature, as the multi-strain probiotic of one or preferentially more genera was superior in treating antibiotic-associated diarrhea in children, growth performance in broilers, protection against *S.* Typhimurium infection in mice, or clearance of *E. coli* O157:H7 from lambs [73]. Our study found that synbiotics have the potential to improve the microbial status of feed for swine, and dietary supplementation of synbiotics decreases the microbial load of muscles, liver, and kidney of pigs. Therefore, the application of synbiotics as a new method aimed at reducing contaminants in feed and food-animal production is beneficial.

According to the authors, the main limitation of the study is the lack of comparative data in the literature on the effect of synbiotics on the feed microbiome. There is also relatively little data on the effect of synbiotics on the microbial load of edible animal raw materials. The small number of animals per group and the shared maternal origin of piglets constitute methodological limitations that may diminish the effective number of independent experimental units. Therefore, the present results should be interpreted as indicative of biologically relevant trends rather than precise population-level estimates, and further studies with larger and fully independent experimental designs are warranted. Despite these limitations, the study provided valuable and pioneering information in its field.

## 5. Conclusions

Synbiotic supplementation reinforces a balance of intestinal flora due to microbial diversity and stability, and interactions with the enteric epithelium as well as the immune system. Our study reveals that synbiotics significantly affected the feed microbiome and reduced general hygienic indicators in edible pork raw materials. A notable finding was the inverse correlation, where high levels of LAB in the feed and feces of groups A, B, and C correlated with a significantly lower LAB count in the animal raw materials, suggesting enhanced intestinal barrier integrity and reduced microbial translocation. Additionally, multi-strain synbiotics showed greater effectiveness in reducing microbial loads compared to single-strain preparations, aligning with the concept of interspecies synergy. The findings suggest that the tested synbiotics are a viable approach to reducing the microbial burden of pork raw materials under standard production conditions, which can help decrease the risk of human infections from contaminated food of animal origin.

## Figures and Tables

**Table 1:** Feed ingredients and chemical composition of the basal diet [15].

Ingredient (g/kg)	Lactating Sow	Prestarter (2–8 Weeks)	Starter (8–12 Weeks)	Grower (12–18 Weeks)	Finisher (12–24 Weeks)
Oats	100	-	-	-	150
Barley	250	220	370	125	200
Triticale	110	-	-	544	361
Wheat	362	300	400	125	150
Soybean meal	90	50	-	100	110
Soybean oil	10	20	20	15	5
Post-extraction soybean meal, heat-treated	40	25	140	-	-
Soybean, full-fat, heat-treated	-	50	-	-	-
Rapeseed meal	-	-	-	60	-
Whey permeate	-	50	-	-	-
Monocalcium phosphate	2	-	-	-	-
LonoFish ^a^	-	50	25	-	-
Specilac ^b^	-	40	-	-	-
Lonacid Max ^c^	-	5	4	1	-
LonoGrain ^d^	-	150	-	-	-
Mycofix PLUS ^e^	-	-	1	-	-
Vitamin-mineral-amino acid premix ^1^	36	-	-	-	-
Vitamin-mineral-amino acid premix ^2^	-	40	-	-	-
Vitamin-mineral-amino acid premix ^3^	-	-	40	-	-
Vitamin-mineral-amino acid premix ^4^	-	-	-	30	25
Chemical composition					
Metabolizable energy (MJ/kg)	13.1	13.8	13.8	13.6	13.5
Crude protein (%)	16.5	18.8	17.9	16.8	15.6
Lysine (%)	0.88	1.56	1.28	1.05	0.95
Methionine + Cysteine (%)	0.59	0.82	0.76	0.66	0.57
Threonine (%)	0.58	0.87	0.79	0.67	0.56
Tryptophan (%)	0.20	0.30	0.20	0.19	0.18
Valine (%)	0.76	0.75	0.79	0.75	0.71
Calcium (%)	1.03	0.91	0.86	0.68	0.67
Phosphorus (%)	0.47	0.61	0.55	0.47	0.38
Vitamin A (IU/kg)	12,500	14,000	20,500	12,000	7700
Vitamin D_3_ (IU/kg)	2000	2000	2000	2000	1540
Vitamin E (mg/kg)	80	84	100	63	100

^a^ protein source; ^b^ feed supplement rich in protein-lactose; ^c^ a dry mixture of phosphoric acid, formic acid, propionic acid, lactic acid, citric acid, acetic acid, and benzoic acid; ^d^ micronized wheat, barley, and maize; ^e^ toxin deactivator; ^1^ complementary feed (4%) L.K. T.CH. (Cargill Poland Sp. z o. o., Warsaw, Poland), Cargill Poland; ^2^ complementary feed (4%) PRESTART. T.CH. (Cargill Poland Sp. z o. o., Warsaw, Poland); ^3^ complementary feed (4%) START. T.CH. (Cargill Poland Sp. z o. o., Warsaw, Poland); ^4^ complementary feed (3/2.5%) GROW/FIN (Cargill Poland Sp. z. o. o, Warsaw, Poland).

**Table 2:** Feed additive composition, dosage, and supplementation scheme [15].

Group	Feed Additive	Composition	Dose	Period of Supplementation (Sows)	Period of Supplementation (Growing Pigs)
A	Synbiotic A	*L. reuteri* ŁOCK 1092 *L. plantarum* ŁOCK 0860 *L. pentosus* ŁOCK 1094 *S. cerevisiae* ŁOCK 0118 Inulin	0.5 kg/tonne of basal diet	10 days before farrowing until weaning	from 2 weeks of age until slaughter
B	Synbiotic B	*L. reuteri* ŁOCK 1092 *L. plantarum* ŁOCK 0860 *L. pentosus* ŁOCK 1094 *S. cerevisiae* ŁOCK 0118 *L. rhamnosus* ŁOCK 1087 Inulin			
C	Synbiotic C	*L. reuteri* ŁOCK 1092 *L. plantarum* ŁOCK 0860 *L. pentosus* ŁOCK 1094 *S. cerevisiae* ŁOCK 0118 *L. rhamnosus* ŁOCK 1087 *L. paracasei* ŁOCK 1091 Inulin			
D	BioPlus_2B^®^	*B. licheniformis* DSM 5749 *B. subtilis* DSM 5750 calcium carbonate kieselguhr as anticaking agent			
E	Cylactin^®^ LBC ME10	*E. faecium* NCIMB 10415 saccharose as carrier cellulose derivative as encapsulating agent			
K	NA	NA	NA	NA	NA

NA—not applicable.

**Table 3:** Standard culture methods used in the study.

No and Title of Method		Step of Method			
		Primary enrichment	Secondary enrichment	Isolation	Confirmation
		liquid non-selective medium/incubation conditions	selective liquid medium/incubation conditions	selective agar medium/incubation conditions	Test
PN-EN ISO 6579:2003 Horizontal method for the detection of *Salmonella* spp. [32]		Buffered Peptone Water ^1^/37 °C/18 h	Rappaport–Vassiliadis soya peptone broth (RVS) ^1^/41.5 °C/24 h	Xylose lysine deoxycholate agar (XLD) ^2^/37 °C/24 h	biochemical identification, serological identification
			Muller–Kauffmann tetrathionate-novobiocin broth (MKTTn) ^2^/37 °C/24 h	Brilliant green agar (BGA) ^2^/37 °C/24 h	
PN-EN ISO 4833-1:2013-12 Horizontal method for the enumeration of microorganisms. Part 1. Colony count at 30 °C by the pour plate technique [33]		NA	NA	Plate count agar (PCA) ^2^/30 °C/72 h	NA
PN-R-64791:1994 Animal feedingstuffs. Requirements and microbiological examination [34]	presence of anaerobic spore-forming bacteria (*Clostridium*)	NA	Wrzosek broth ^3^/37 °C/48 h/anaerobic conditions	Willis-Hobbs agar ^3^/37 °C/48 h/ anaerobic conditions	biochemical identification, microscopic identification (Gram staining)
				Wilson-Blair agar ^2^/37 °C/48 h/ anaerobic conditions	
	enumeration of fungi	NA	NA	Dichloran-rose Bengal chloramphenicol agar (DRBC) ^2^/25 °C/7 d	NA
	enumeration of aerobic mesophilic bacteria	NA	NA	Nutrient agar ^2^/37 °C/48 h	NA
PN-EN ISO 11290-1:1999/A1:2004 Horizontal method for the detection and enumeration of *Listeria monocytogenes*. Part 1. Detection method [35]		half-Fraser broth ^2^/37 °C/24 h	Fraser broth ^2^/37 °C/24 h	ALOA agar ^4^/37 °C/24 h	biochemical identification
				Oxford agar ^2^/37 °C/24 h	
PN-EN ISO 7937:2005 Horizontal method for the enumeration of *Clostridium perfringens*. Colony-count technique [36]		NA	NA	Tryptose sulfite cycloserine agar (SC) ^2^/37 °C/20 h/a anaerobic conditions	biochemical identification
PN-ISO 21528-2:2005 Horizontal method for the detection and enumeration of Enterobacteriaceae. Part 2. Colony-count technique [37]		NA	NA	Violet red bile glucose agar (VRBG) ^1^/37 °C/24 h	biochemical identification
PN-ISO 16649-2:2004 Horizontal method for the enumeration of beta-glucuronidase-positive *Escherichia coli*. Part 2. Colony-count technique at 44 °C using 5-bromo-4-chloro-3-indolyl beta-D-glucuronide [38]		NA	NA	Tryptone bile x-glucuronide agar (TBX) ^2^/44 °C/24 h	NA
PN-EN ISO 6888-2:2001 Horizontal method for the enumeration of coagulase-positive staphylococci (*Staphylococcus aureus* and other species). Part 2: Technique using rabbit plasma fibrinogen agar medium [39]		NA	NA	Baird-Parker RPF agar ^2^/37 °C/24–48 h	NA
PN-A-82055-12:1997 Detection of anaerobic spore-forming bacteria and anaerobic spore-forming sulfate(IV)-reducing bacteria [40]		NA	Wrzosek broth ^3^/37 °C/48 h/anaerobic conditions	Willis-Hobbs agar ^3^/37 °C/48 h/anaerobic conditions	biochemical identification, microscopic identification (Gram staining)
				Wilson-Blair agar ^2^/37 °C/48 h/anaerobic conditions	
PN-EN ISO 10272-1:2007 Horizontal method for detection and enumeration of *Campylobacter* spp. Part 1: Detection method [41]		NA	Bolton selective enrichment broth ^2^/41.5 °C/48 h/microaerobic conditions	Modified charcoal-cefoperazone-deoxycholate agar (mCCDA) ^2^/41.5 °C/48 h/microaerobic conditions	biochemical identification, morphological identification
PN-A-82055-7:1997 Detection and enumeration of enterococci [42]		NA	NA	Slanetz and Bartley agar ^2^/37 °C/24–48 h	microscopic identification, biochemical identification, morphological identification
PN-EN 15788:2009 Isolation and enumeration of *Enterococcus* (*E. faecium*) spp. [43]		NA	NA	Bile esculin azide agar (BEAA) ^2^/37 °C/24 h	microscopic identification, morphological identification
PN-ISO 15214:2002 Horizontal method for the enumeration of mesophilic lactic acid bacteria. Colony-count technique at 30 °C [44]		NA	NA	De Man, Rogosa and Sharpe agar (MRS) ^2^/30 °C/72 h	NA
PN-EN 15789:2009 Isolation and enumeration of yeast probiotic strains [45]		NA	NA	Chloramphenicol glucose yeast extract agar (CGYE) ^2^ /35 °C/48 h	microscopic identification, morphological identification
PN-EN ISO 7932:2005 Horizontal method for the enumeration of presumptive *Bacillus cereus*. Colony-count technique at 30 °C [46]		NA	NA	Mannitol yolk polymyxin agar (MYP) ^2^/30 °C/24 h	biochemical identification

NA—not applied, d—days, h—hours, ^1^ Merck KGaA, Darmstadt, Germany; ^2^ Oxoid Ltd., Basingstoke, UK; ^3^ BTL Sp. z o.o., Lodz, Poland; ^4^ Bio-Rad Laboratories, Inc., Hercules, USA.

**Table 4:** Microbial status of negative controls of feed and animal raw materials.

Microorganism	Feed			Animal Raw Material	
	Sow	Piglet	Fattener	Piglet	Fattener
*Salmonella* spp. (%)	0	0	0	0	0
*Campylobacter* spp. (%)	0	0	0	0	0
*L. monocytogenes* (%)	0	0	0	0	0
*Clostridium* spp. (%)	66.6	66.6	66.6	11.1	0
*C. perfringens* (%)	33.3	33.3	33.3	11.1	0
*C. botulinum* (%)	0	0	0	0	0
TPC (log_10_ cfu/g)	6–7.3	5.3–6	5.6–6.6	<1–5	<1–5
AMB (log_10_ cfu/g)	6–6.7	5.2–5.8	5.3–6.7	<1–5.1	<1–4.8
Fungi (log_10_ cfu/g)	5.5–6.1	5.3–5.5	4.3–5.5	<1	<1–3.5
Enterobacteriaceae (log_10_ cfu/g)	5.3–6.2	4.1–4.6	4.7–5.9	<1	<1–2.9
*E. coli* (log_10_ cfu/g)	<1–1.6	<1	<1	<1	<1–2.7
*Clostridium* spp. (log_10_ cfu/g)	<1–2	<1–4	<1–4	<1–2	<1
*C. perfringens* (log_10_ cfu/g)	<1	<1	<1	<1	<1
*B. cereus* (log_10_ cfu/g)	<1	<1	<1	<1	<1
CoPS (log_10_ cfu/g)	<1	<1	<1	<1	<1
HS (log_10_ cfu/g)	<1	<1	<1	<1	<1
LAB (log_10_ cfu/g)	<1–4.2	<1	<1	<1–2.8	<1–3.3
*Enterococcus* (log_10_ cfu/g)	2.9–3.9	<1–5.5	<1–2.2	<1	<1
Yeast probiotic strains (log_10_ cfu/g)	<1	<1–4.1	<1	<1	<1

**Table 5:** Prevalence (% and 95% CI) of microorganisms in feed and animal raw materials.

Microrganisms	Group of Animals					
	A	B	C	D	E	K
	Feed					
*Salmonella* spp.	0.0 (0.0–33.6)	0.0 (0.0–33.6)	0.0 (0.0–33.6)	0.0 (0.0–33.6)	0.0 (0.0–33.6)	0.0 (0.0–33.6)
*Campylobacter* spp.	0.0 (0.0–33.6)	0.0 (0.0–33.6)	0.0 (0.0–33.6)	0.0 (0.0–33.6)	0.0 (0.0–33.6)	0.0 (0.0–33.6)
*Listeria* spp.	22.2 (2.8–60.0)	11.1 (0.3–48.2)	22.2 (2.8–60.0)	11.1 (0.3–48.2)	0.0 (0.0–33.6)	0.0 (0.0–33.6)
*L. monocytogenes*	0.0 (0.0–33.6)	0.0 (0.0–33.6)	0.0 (0.0–33.6)	0.0 (0.0–33.6)	0.0 (0.0–33.6)	0.0 (0.0–33.6)
*Clostridium* spp.	66.7 (29.9–92.5)	88.9 (51.8–99.7)	77.8 (40.0–97.2)	88.9 (51.8–99.7)	88.9 (51.8–99.7)	66.7 (29.9–92.5)
*C. perfringens*	55.6 (21.2–86.3)	33.3 (7.5–70.1)	88.9 (51.8–99.7)	88.9 (51.8–99.7)	11.1 (0.3–48.2)	33.3 (7.5–70.1)
*C. botulinum*	0.0 (0.0–33.6)	0.0 (0.0–33.6)	0.0 (0.0–33.6)	0.0 (0.0–33.6)	0.0 (0.0–33.6)	0.0 (0.0–33.6)
	Animal raw materials					
*Salmonella* spp.	0.0 (0.0–12.8)	0.0 (0.0–12.8)	0.0 (0.0–12.8)	0.0 (0.0–12.8)	0.0 (0.0–12.8)	0.0 (0.0–12.8)
*Campylobacter* spp.	0.0 (0.0–12.8)	0.0 (0.0–12.8)	0.0 (0.0–12.8)	0.0 (0.0–12.8)	0.0 (0.0–12.8)	0.0 (0.0–12.8)
*Listeria* spp.	0.0 (0.0–12.8)	0.0 (0.0–12.8)	3.7 (0.1–19.0)	0.0 (0.0–12.8)	0.0 (0.0–12.8)	0.0 (0.0–12.8)
*L. monocytogenes*	0.0 (0.0–12.8)	0.0 (0.0–12.8)	3.7 (0.1–19.0)	0.0 (0.0–12.8)	0.0 (0.0–12.8)	0.0 (0.0–12.8)
*Clostridium* spp.	7.4 (0.9–24.3)	0.0 (0.0–12.8)	0.0 (0.0–12.8)	0.0 (0.0–12.8)	3.7 (0.1–19.0)	3.7 (0.1–19.0)
*C. perfringens*	7.4 (0.9–24.3)	0.0 (0.0–12.8)	0.0 (0.0–12.8)	0.0 (0.0–12.8)	3.7 (0.1–19.0)	3.7 (0.1–19.0)
*C. botulinum*	0.0 (0.0–12.8)	0.0 (0.0–12.8)	0.0 (0.0–12.8)	0.0 (0.0–12.8)	0.0 (0.0–12.8)	0.0 (0.0–12.8)

## Data Availability

The raw data supporting the conclusions of this article will be made available by the authors on request.
